# Koninginins X-Z, Three New Polyketides from *Trichoderma koningiopsis* SC-5

**DOI:** 10.3390/molecules28237848

**Published:** 2023-11-29

**Authors:** Weiwei Peng, Jianbing Tan, Zihuan Sang, Yuantao Huang, Li Xu, Yuting Zheng, Siyu Qin, Haibo Tan, Zhenxing Zou

**Affiliations:** 1 Xiangya School of Pharmaceutical Sciences, Central South University, Changsha 410013, China; pww199802@163.com (W.P.); tanjb1009@csu.edu.cn (J.T.); sangzihuan123@163.com (Z.S.); 217211012@csu.edu.cn (L.X.); 217211011@csu.edu.cn (Y.Z.); siyuqin1226@163.com (S.Q.); 2 Hunan Key Laboratory of Diagnostic and Therapeutic Drug Research for Chronic Diseases, Changsha 410013, China; 3 Key Laboratory of South China Agricultural Plant Molecular Analysis and Genetic Improvement, Guangdong Provincial Key Laboratory of Applied Botany, South China Botanical Garden, Chinese Academy of Sciences, Guangzhou 510520, China; 4 Affiliated Haikou Hospital of Xiangya School of Medicine, Central South University, Haikou 570208, China; hyt951232@163.com

**Keywords:** *Trichoderma koningiopsis*, endophytic fungi, secondary metabolites, polyketide, antifungal activity

## Abstract

Koninginins X-Z (**1**–**3**), three novel polyketides, were isolated from the solid fermentation of the endophytic fungus *Trichoderma koningiopsis* SC-5. Their structures, including the absolute configurations, were comprehensively characterized by a combination of NMR spectroscopic methods, HRESIMS, ^13^C NMR, DFT GIAO ^13^C NMR, and electronic circular dichroism calculations as well as single crystal X-ray diffraction. In addition, all the compounds were evaluated for antifungal activity against *Candida albicans*.

## 1. Introduction

Plant pathogens are ubiquitous microorganisms that have profound impacts on the agricultural, industrial, pharmaceutical, and medical domains [1]. Beyond causing crop diseases, they play a dual role by also serving as biological control agents in agriculture, safeguarding crops [2,3]. Their unique characteristics enable applications in biofuel and industrial chemical production, while their secondary metabolites, rich in biological activities, hold promise for new drug developments in medicine [4,5]. The genus *Trichoderma*, a widely distributed fungal group in nature, mainly exists in the ecological environment of plant inter-root soil, foliage, seeds, and bulbs. It stands as the most extensively researched and utilized phytopathogenic antagonist fungus among biopreventive fungi employed for plant disease control [6,7]. Notably, *Trichoderma* is one of the best-represented plant antagonistic fungi, contributing to biological control and the discovery of new natural products, such as polyketides, alkaloids, and terpenoids [8,9,10,11,12]. Koninginin derivatives, a kind of typical polyketides produced by the species of the *Trichoderma* genus, most of which share an intriguing bicyclic pyran skeleton with a characteristic hemiketal or ketal moiety [13,14,15,16,17,18,19,20,21,22], have successfully aroused our keen interest in medicinal discoveries.

**Figure 1:**
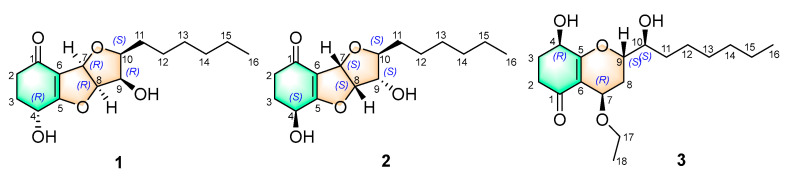
Structures of compounds **1**–**3**.

During our continuing search for novel microbial natural products [23,24,25,26,27], we have previously reported two novel koninginin derivatives sharing unprecedented nitrogenous fused polycyclic polyketide skeletons from *Trichoderma koningiopsis* SC-5 [28]. With the examination of other similar structurally unique and biologically meaningful koninginins, the further chemical investigation of *Trichoderma koningiopsis* SC-5 successfully led to the isolation of three novel koninginin derivatives, koninginins X-Z (**1**–**3**) (Figure 1). Herein, the isolation, structural elucidation, and antifungal activity evaluation of these three isolates are described in detail.

## 2. Results and Discussion

Koninginin X (**1**) was isolated as a yellow oil, HRESIMS-(+) analysis of molecular ion peaks at *m/z* 297.1703 [M + H]^+^ (calcd. for C_16_H_25_O_5_^+^, 297.1702) and 319.1523 [M + Na]^+^ (calcd. for C_16_H_24_O_5_Na^+^, 319.1521) collectively pointed to the molecular formula C_16_H_24_O_5_, requiring 5 indices of hydrogen deficiency (IHDs). Its emblematic NMR spectroscopic signals (Table 1) of five oxygenated methines at *δ*_C/H_ 64.0/4.75 (C-4), 79.4/5.25 (C-7), 89.5/5.25 (C-8), 72.0/4.35 (C-9), 82.1/3.66 (C-10) and a terminal methyl group at *δ*_C/H_ 14.0/0.87 (C-16) together with a carbonyl functionality at *δ*_C_ 194.0 (C-1) and two olefinic carbons at *δ*_C_ 178.5 (C-5), 115.1 (C-6) were distinctively recognized by holistic analysis of ^1^H, ^13^C NMR, DEPT 135, and HSQC spectra. The established functionalities of **1** chemo-logically accounted for two IHDs and strongly suggested the chemical structure of **1** to be a tricyclic skeleton.

**Figure 2:**
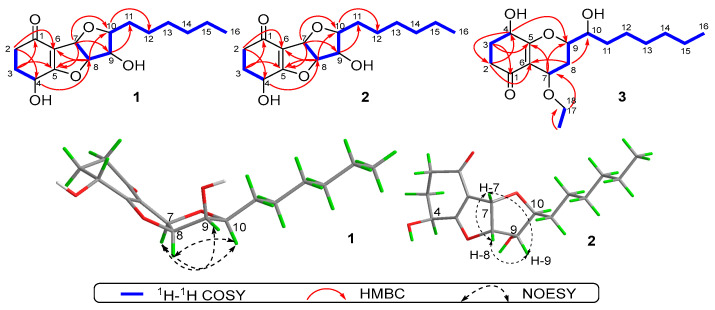
^1^H-^1^H COSY, key HMBC, and NOESY correlations of **1**–**3**.

The planar structure of **1** was established by a thorough analysis of its ^1^H-^1^H COSY and HMBC spectra (Figure 2). Firstly, two spin-spin coupling segments of H_2_-2/H_2_-3/H-4 and H-7/H-8/H-9/H-10/H2-11/H2-12/H2-13/H2-14/H2-15/H3-16 were observed in the ^1^H-^1^H COSY spectrum. Then, the critical HMBC correlations from H_2_-2 to C-1/C-3/C-4/C-6, H_2_-3 to C-1/C-2/C-4/C-5, H-4 to C-2/C-6, and the COSY cross peaks of H_2_-2/H_2_-3/H-4 absolutely constructed the ring system of cyclohexanone. In addition, the HMBC correlations from H-7 to C-5/C-9/C-10, H-8 to C-5/C-6/C-10, together with the remaining two IHDs, necessitated C-5/C-6/C-7/C-8/C-9/C-10 to form a double-furan fused ring system. Therefore, the planar structure of **1** was completely illustrated, and it was recommended to share an identical framework with that of trichodermaketone B [9]. Similarly, an obvious negative Cotton effect at 286 nm (∆*ε* − 3.26) was observed in the CD spectrum of **1**, which informatively suggested the absolute configuration to be 4*R* [9]. In addition, compound **1** possessed the same relative configuration as that of trichodermaketone B, referring to the key NOE correlations (Figure 2), while the absolute configuration has not been completely identified.

**Figure 3:**
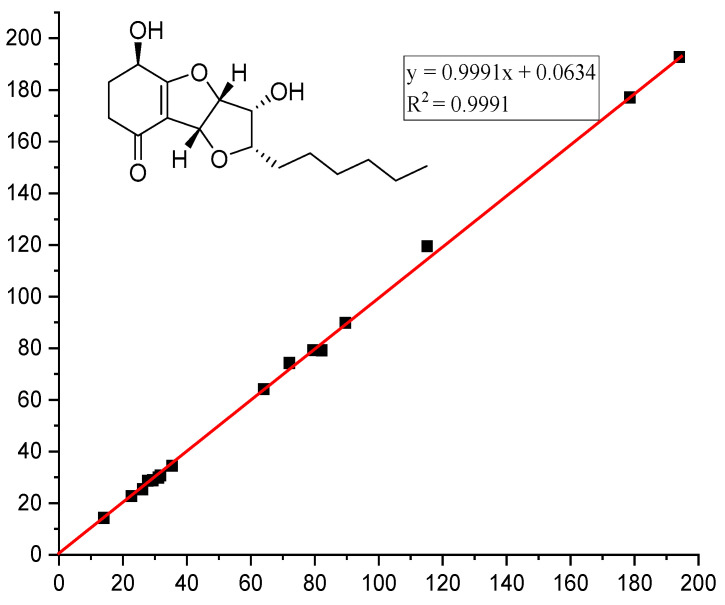
Regression analyses of experimental and calculated ^13^C NMR chemical shifts of **1**.

**Figure 4:**
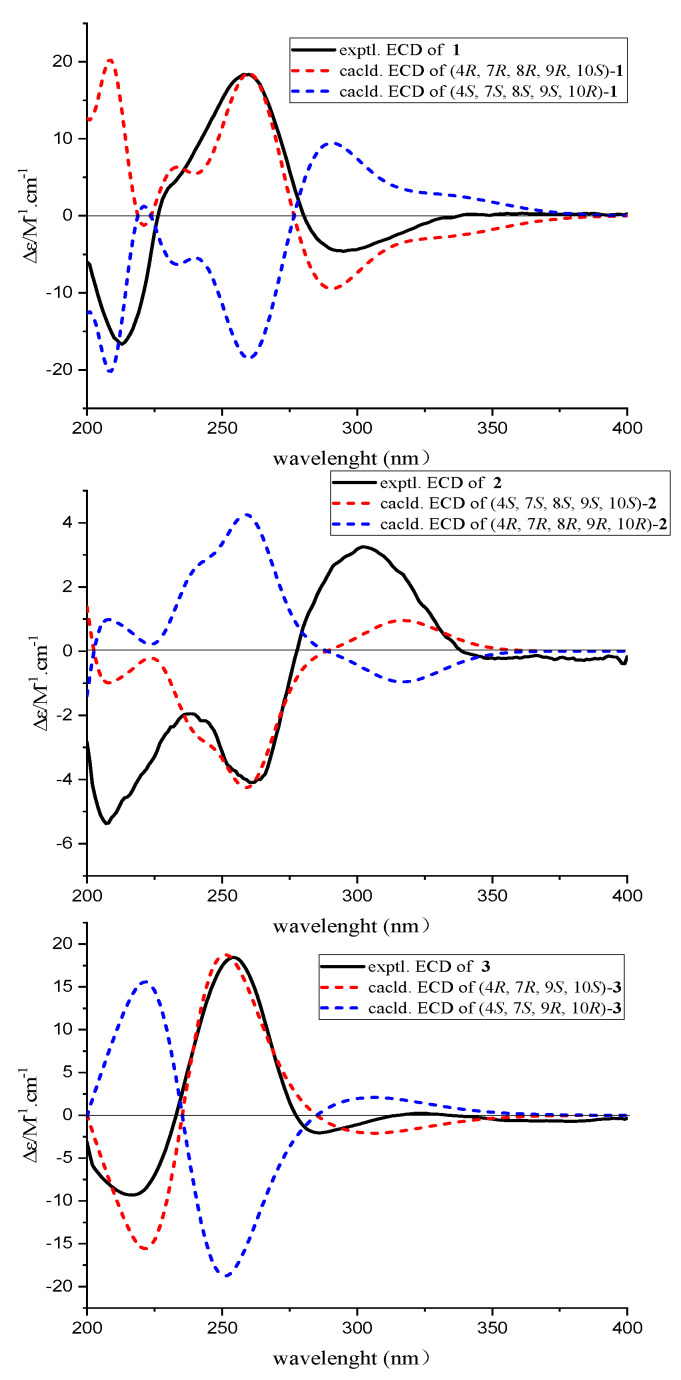
Experimental and calculated ECD spectra of **1**–**3** (in MeOH).

The chemical structure of **1** was further affirmed by ^13^C NMR calculation, and the ^13^C NMR calculated data for the 4R*,7R*,8R*,9R*,10S*-**1** conformer showed a correlation coefficient (*R^2^*) of 0.9991 (Figure 3) and a small mean absolute error (MAE) of 1.15. Moreover, a minute root mean square (RMS) of 1.60 and the critical *Prel* value of 100% were also obtained for the 4R*,7R*,8R*,9R*,10S*-**1** conformer, which thus further evidenced the probability of the calculated result (Table 2). Subsequently, we determined the absolute configuration of **1** by theoretical ECD calculation, and its absolute configuration was finally revealed to be 4*R*,7*R*,8*R*,9*R*,10*S*, as depicted in Figure 4. Consequently, the absolute configuration of **1** was absolutely elucidated and given a trivial name, (4*R*,7*R*,8*R*,9*R*,10*S*)-koninginin X.

Compound **2** was gathered as a stereoisomer of **1** and had the same molecular formula, C_16_H_24_O_5_, determined by HRESIMS-(+) analysis (*m*/*z* 297.1710 [M + H]^+^ and 319.1530 [M + Na]^+^). The NMR data (Table 1) of **2** and key ^1^H-^1^H COSY and HMBC correlations (Figure 2) also demonstrated that both compounds should share the same planar structure. However, according to the ^13^C NMR spectrum, the chemical shift of C-2 at *δ*_C_ 35.6 in trichodermaketone B was upshifted to *δ*_C_ 22.5 in **2**, while the chemical shift of C-4 at *δ*_C_ 63.6 in trichodermaketone B was downshifted to *δ*_C_ 71.7 in **2**; the aforementioned informative results tentatively indicated the relative configuration of C-4 of **2** might differ from that of the known trichodermaketone B [9,29,30]. Interestingly, the CD experiment of **2** showed a clear positive Cotton effect for the n → π* transition at 302 nm (∆*ε* + 3.25), which further suggested the absolute configuration for **2** to be 4*S* and consolidated the aforementioned deduction. Notably, the 4*S* configuration of **2** was absolutely opposite to 4*R* for the known compounds, trichodermaketones A and B [9].

Furthermore, in the NOE difference experiments (Figure 2), the irradiation of H-8 (*δ*_H_ 5.05) led to the enhancement of H-7 (*δ*_H_ 5.65) and H-9 (*δ*_H_ 4.16), the irradiation of H-7 (*δ*_H_ 5.65) led to the enhancement of H-8 (*δ*_H_ 5.05) and H-9 (*δ*_H_ 4.16), and the irradiation of H-10 (*δ*_H_ 3.43) resulted in the enhancement of H-9 (*δ*_H_ 4.16). Based on these NOE signals, the chiral protons of H-7, H-8, and H-9 were then suggested to be on the same side, while H-10 should be on the other orientation. Therefore, the relative configuration of **2** was completely established.

Subsequently, with the aim to verify the relative configuration and determine the absolute configuration of **2**, the time-dependent density functional (TDDFT)-ECD calculations in Gaussian 16 were applied. In Figure 4, the calculated ECD curve of 4*S*,7*S*,8*S*,9*S*,10*S*-2 was consistent with that of the experimental one by a close comparison, thus ambiguously suggesting that the absolute configuration of **2** was 4*S*,7*S*,8*S*,9*S*,10*S*. As a result, the structure of **2** was fully determined and given the trivial name, (4*S*,7*S*,8*S*,9*S*,10*S*)-koninginin Y.

Compound **3** was obtained as a yellow oil; its molecular formula was confirmed as C_18_H_30_O_5_ on the basis of the positive HRESIMS ion peak at m/z 349.1989 [M + Na]^+^ (calcd. for C_18_H_30_O_5_Na^+^, 349.1991), which required four degrees of unsaturation. Comprehensive analyses of the 1D and 2D NMR spectra for **3** disclosed the presence of two methyl groups [*δ*_C/H_ 14.6/0.91 (C-16) and 16.0/1.15 (C-18)], nine methylenes [*δ*_C/H_ 34.4/2.63, 2.29 (C-2), 30.6/2.18, 1.97 (C-3), 29.3/2.03, 1.55 (C-8), 33.8/1.64 (C-11), 26.8/1.55, 1.41 (C-12), 30.6/1.41, 1.33 (C-13), 33.2/1.33 (C-14), 23.8/1.33 (C-15), and 65.4/3.64, 3.55 (C-17)], four methines [*δ*_C/H_ 67.1/4.38 (C-4), 65.4/4.35 (C-7), 78.2/4.14 (C-9), and 73.6/3.68 (C-10)], and three non-proton carbons with the formation of *α*,*β*-unsaturated ketone units [*δ*_C_ 113.2 (C-6), 174.9 (C-5), and 199.4 (C-1)].

Then, a detailed comparison of the NMR data of **3** with those of 7-O-methylkoninginin D [9], a koninginin derivative isolated from the marine fungus *Trichoderma koningii*, implied that **3** should also be a closely similar derivative of koninginin D. The NMR data for both compounds are very similar, excepting that the methoxy group [*δ*_C_ 59.6 (C-17)] in 7-O-methylkoninginin D was changed to be an ethoxy group [*δ*_C_ 65.4 (C-17) and *δ*_C_ 16.0 (C-18)] in **3**. Moreover, the cross peak between H_2_-17 and H_3_-18 in the ^1^H-^1^H COSY spectrum, together with the critical HMBC correlations from H_3_-18 to C-17 and H_2_-17 to C-7, further strengthened the presence of the ethoxy functional group and suggested its location at the C-7 position. Moreover, other observed HMBC correlations and ^1^H-^1^H COSY cross peaks (Figure 2) further established the planar structure of **3**.

Nevertheless, the relative configuration of compound **3** was unable to be established by the NOESY experiment, because there were no diagnosable correlative signals obviously observed, and the configuration of 7-O-methylkoninginin D was previously determined from an inference of biogenetic perspective [9]. Then, the *J* values of ^1^H NMR were used to determine the relative configuration of **3** [29,30,31]. In the ^1^H NMR spectrum, the coupling constants for H-7/H-8*α* with *J* = 2.4 Hz, H-8*α*/H-8*β* with *J* = 14.4 Hz, and H-9/H-8*β* with *J* = 2.4 Hz were essentially located H-7 and H-9 in a trans-diequatorial relationship. Additionally, the coupling constant (*J* = 12.6 Hz) for H-10/H-9 also indicated H-9 and H-10 in a trans-diequatorial relationship. Moreover, a negative Cotton effect at 286 nm (∆*ε* − 2.05) for the n → π* transition in the CD spectrum of **3** also suggested the absolute configuration of 4*R* [9,32].

**Figure 5:**
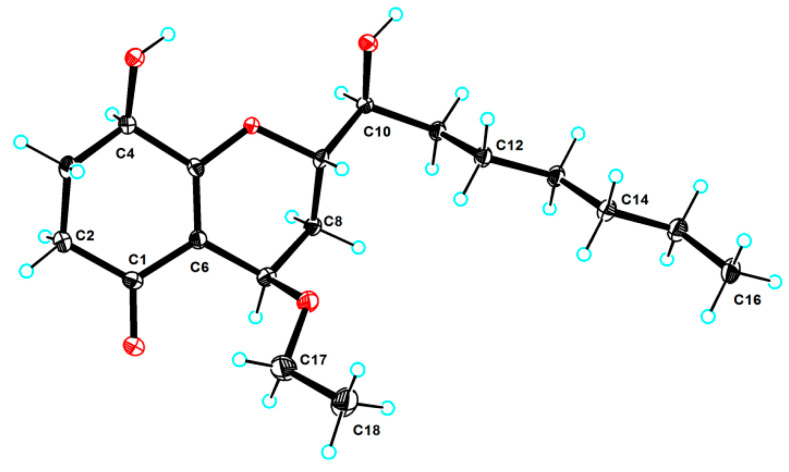
ORTEP drawing of the X-ray structure of **3**.

Subsequently, the absolute configuration of **3** was further confirmed by comparison of the experimental and the simulated circular dichroism (CD) spectra. As shown in Figure 4, the calculated ECD curve of 4*R*,8*R*,9*S*,10*S*-**3** was perfectly matched with the experimental one, thus strongly illustrating that the absolute configuration of **3** was 4*R*,8*R*,9*S*,10*S*. Satisfactorily, the absolute configuration of **3** with 4*R*,8*R*,9*S*,10*S* was further ambiguously verified by single crystal X-ray diffraction with a Flack parameter of 0.15 (11) (Figure 5), Therefore, the absolute configuration of compound **3** was completely determined and given the trivial name, (4*R*,8*R*,9*S*,10*S*)-koninginin Z.

Conclusively, the type of chemical structure for the koninginin family was first reported in 1989 [33]; nowadays, it has been increasingly arousing the great interest of many pharmacists with an aim to discover novel lead drugs with structurally fascinating skeletons and biologically significant activities [10,17,21], which thus boosts the reporting of novel compounds in the koninginin family [9,10,11,12,28]. However, the intractable challenge and imperfect issue clouding the koninginin family is that the configurations for many chemical structures had not been thoroughly solved [9,18,21]. In this research, the chemical structures, including the absolute configuration of these novel isolates, were comprehensively characterized with the help of the combination of NMR spectroscopic methods, HRESIMS, electronic circular dichroism calculations, and single crystal X-ray diffraction. We believe that these reliable methodologies towards efficient structure resolution in this study could act as a promisingly applicable strategy to resolve the configuration determination of other koninginin derivatives.

Notably, the genus *Trichoderma* has been revealed in recent years to be a high producer of natural products [34]. Polyketides are the critical characteristic chemical constituents for the genus *Trichoderma* and reported extensively. They usually share a variety of intriguing structures and novel carbon-chain skeletons [34]. As exemplified by the sorbicillinoid-based compounds, saturnispols A-H, all of them share a novel cyclic hexaketide nucleus and a typical sorbyl sidechain in their chemical scaffold [35]. Moreover, trichorenins A-C with a unique tetracyclic carbon skeleton were also discovered from the genus *Trichoderma* [36], and tandyukisins G-I possess an attractive chromone core with a natural rarely occurring 4-oxo-4H-1-benzopyran scaffold representing a class of unprecedented polyketides that are widely distributed in *Trichoderma* sp. [37]. Although the genus *Trichoderma* has been widely studied due to the diversity of natural environmental systems, the genus *Trichoderma* parasitized by different biological groups is likely to produce more natural products with novel structures and significant biological activities under the influence of an environmental host and their own evolution [35,38,39]. Therefore, the genus *Trichoderma* is a significantly promising strategic bio-resource for the excavation of structurally novel lead natural products, and further extensive research efforts on their chemical constituents are still called upon for their great research value and spacious application prospect.

In the previously reported literature [18,19,40], the natural products derived from the genus *Trichoderma* usually exhibit considerable antifungal activity against a variety of different pathogenic fungi. For example, trichodermatide B from *Emericella nidulans* showed significant antifungal activity against *Cryptococcus neoformans*, with an IC_50_ value of 4.9 μg/mL [37]. Therefore, compounds **1**–**3** were further evaluated for antifungal activity against *Candida albicans*, where they did not exhibit any notable antifungal effect at the concentration of 100 μg/mL. Notably, we evaluated the antibacterial and cytotoxic activities of the isolated koninginin compounds from *Trichoderma koningiopsis* SC-5 in our previous experiments [28], and none of them showed any significant activity. In the future, more pathogenic fungi should be considered to evaluate the antifungal effects of these koninginin compounds, and many other biological activities such as antiviral and anti-inflammatory activities also worthy of testing.

## 3. Materials and Methods

### 3.1. General Experimental Procedures

The HRESIMS analyses were carried out on an Agilent 6500 series Q-TOF mass spectrometer (Agilent, Singapore) in the positive ion mode. The 1D and 2D NMR data were measured on a Bruker AVIII-600 spectrometer (Bruker, Karlsruhe, Germany) using TMS as an internal standard. Optical rotations were recorded on an Anton Paar MCP-500 spectropolarimeter (Anton Paar, Graz, Austria). Experimental ECD spectra were acquired from Applied PhotoPhysics’ Chiascan circular dichroism spectrometer (APL, Surrey, UK). The single crystal data were collected on an Agilent Xcalibur Novasingle crystal diffractometer equipped with CuK*α* radiation. The reversed-phase preparative HPLC was conducted using an Agilent 1100 (Agilent Technologies, Santa Clara, CA, USA) instrument connected to an Innoval ODS-2 column (5 μm, 10 mm × 250 mm) with a VWD detector. The UV spectra were recorded with the use of a UV-2600 spectrophotometer (Shimadzu, Kyoto, Japan). Silica gel (Qingdao Marine Chemical Factory, Qingdao, China), macroporous resin (Hebei, China), Sephadex LH-20 (Toyopearl Tosoh, Tokyo, Japan), and octadecylsilane reversed-phase silica gel (50 µm, Fuji, Kasugai, Japan) were utilized for column chromatography. All the solvents were of analytical grade.

### 3.2. Fungal Material

The strain of *Trichoderma koningiopsis* SC-5 was isolated from *Pedicularis integrifolia* in Li County, Aba Tibetan and Qiang Autonomous Prefecture, Sichuan Province. It was collected in July 2021. This endophytic strain was subsequently identified by the sequence analysis of the rDNA ITS region, and the sequence of its rDNA ITS region was submitted to GenBank (Accession: OP646773). This endophytic fungus is preserved in the Department of Medicinal Chemistry, Xiangya School of Pharmaceutical Sciences, Central South University.

### 3.3. Fermentation, Extraction, and Isolation

The fungus was cultured in autoclaved rice solid medium (120 × 500 mL Erlenmeyer flasks, each containing 100 g of grain and 120 mL of ultrapure water). The fermentation was performed under static conditions at a constant temperature of 28 °C for 4 weeks. The fungal culture (5 kg) was extracted with EtOAc (10 L) three times, and the extract was subsequently decompression-concentrated to provide 110.0 g crude products.

The EtOAc extract was subjected to macroporous resin column chromatography (CC) eluting with EtOH-H_2_O (*v*/*v*, 20:80–0:100) to give three fractions (Fr. 1 to Fr. 3). Fr. 2 (45 g) was further separated on a silica gel CC eluting with CH_2_Cl_2_-MeOH (*v*/*v*, 100:1–0:100) to obtain 7 fractions (Fr. 2a to Fr. 2g). Subsequently, Fr. 2d (8.5 g) was separated by a reversed-phase ODS column with a gradient of MeOH-H_2_O (*v*/*v*, 5:95–100:0) to provide 11 subfractions (Fr. 2d-1 to Fr. 2d-11); among them, Fr. 2d-4 was separated by a Sephadex LH-20 column with MeOH-H_2_O (*v*/*v*, 80:20) and was further purified by prep-HPLC with CH_3_CN-H_2_O (0–50 min, 25–50%, 2 mL/min) to yield compound **3** (15.2 mg, t_R_ = 42.5 min). Fr. 2d-3 was separated by a Sephadex LH-20 column with MeOH-H_2_O (*v*/*v*, 80:20), and was further purified by prep-HPLC with CH_3_CN-H_2_O (0–40 min, 25–45%, 2 mL/min) to obtain compound **1** (6.0 mg, t_R_ = 36.2 min) and **2** (2.8 mg, t_R_ = 38.2 min).

Koninginin X (**1**). Yellow oil; ${\left[\alpha \right]}_{D}^{20}$ + 9.32 (c 0.30, MeOH); UV (MeOH): *λ*_max_ (log *ε*): 257 (2.75) nm; ECD (0.36 mg/mL, MeOH): *λ*_max_ (Δ*ε*) 233 (+6.30), 260 (+18.48), 291 (−9.45) nm; ^1^H (CDCl_3_, 600 MHz) and ^13^C (CDCl_3_, 150 MHz) NMR spectral data, see Table 1; HRESIMS: *m*/*z* 297.1703 [M + H]^+^ (calcd. for C_16_H_25_O_5_^+^, 297.1702), 319.1523 [M + Na]^+^ (calcd. for C_16_H_24_O_5_Na^+^, 319.1521).

Koninginin Y (**2**). Yellow oil; ${\left[\alpha \right]}_{D}^{20}$ − 55.14 (c 0.13, MeOH); UV (MeOH): *λ*_max_ (log *ε*): 257 (3.12) nm; ECD (0.36 mg/mL, MeOH): *λ*_max_ (Δ*ε*) 207 (−5.37), 239 (−1.95), 261 (−4.10), 302 (+3.25) nm; ^1^H (CDCl_3_, 600 MHz) and ^13^C (CDCl_3_, 150 MHz) NMR spectral data, see Table 1; HRESIMS: *m*/*z* 297.1710 [M + H]^+^ (calcd. for C_16_H_25_O_5_^+^, 297.1702), 319.1530 [M + Na]^+^ (calcd. for C_16_H_24_O_5_Na^+^, 319.1521).

Koninginin Z (**3**). Yellow oil; ${\left[\alpha \right]}_{D}^{20}$ + 164.87 (c 0.36, MeOH); HPLC-UV (CH_3_CN-H_2_O) *λ*_max_: 258 nm; ECD (0.36 mg/mL, MeOH): *λ*_max_ (Δ*ε*) 217 (−14.21), 254 (+18.21), 306 (−0.74) nm; ^1^H (CD_3_OD, 600 MHz) and ^13^C (CD_3_OD, 150 MHz) NMR spectral data, see Table 1; HRESIMS: *m*/*z* 349.1989 [M + Na]^+^ (calcd. for C_18_H_30_O_5_Na^+^, 349.1991).

### 3.4. X-ray Crystallographic Analysis

Crystal data for **3** C_18_H_30_O_5_ (M = 326.43 g/mol): monoclinic, space group P21 (no. 4), *a* = 5.0609(2) Å, *b* = 39.7106(9) Å, *c* = 9.8073(3) Å, *V* = 1905.11(11) Å3, Z = 2, T = 100.15 K, *μ*(CuK*α*) = 0.759 mm^−1^, *Dcalc* = 1.232 g/cm^3^, 6178 reflections measured (8.908 ≤ 2Θ ≤ 148.692), 6178 unique (*R*_int_ = 0.0298, *R*_sigma_ = 0.0394), which were used in all the calculations. The final *R*_1_ was 0.0423 (I > 2*σ*(I)), and *wR*_2_ was 0.1121 (all data). Flack parameter = 0.15(11). The crystallographic data for **3** reported in this paper was deposited in the Cambridge Crystallographic Data Centre. (Deposition number: CCDC 2264777). Copies of these data can be obtained free of charge via https://www.ccdc.cam.ac.uk (accessed on 18 June 2023).

### 3.5. Quantum Chemistry Calculations

A conformational search was performed by Crest [41], followed by optimization on a GFN2-xTB [42] level with a 4 kcal/mol energy window to remove high-energy conformers. The optimization and frequency calculation of each conformer was implemented on a B3LYP-D3(BJ)/TZVP [43,44] level of theory by using a Gaussian 16 software package with the keyword: g09defaults [45]. The DFT GIAO ^13^C NMR calculation was performed on the *ω*B97x-D/6-31G* level [46], and the data processing followed the reported STS protocol [47]. The ECD calculations were by TDDFT on the *ω*B97x-D/TZVP level of theory and were Boltzmann-averaged according to the Gibbs free energy. SpecDis v1.71 [48] was used to simulate the ECD curves of compounds **1**–**3**, with sigma/gamma values of 0.30 eV, 0.30 eV, and 0.40 eV, respectively. The averaged calculated ECD curves of compounds **1**–**2** were adjusted by blue shifting for 15 nm and 10 nm, respectively.

### 3.6. Biological Activity Assay

The antifungal activity against *Candida albicans* (ATCC 10231) was evaluated by using a broth microdilution protocol modified from the CLSI (formerly NCCLS) M-38A and M-27A2 methods [9]. The compounds and positive drug vancomycin were prepared at a concentration of 100 µg/mL in the growth medium, and the solvent control used DMSO. The MIC value was regarded as the lowest inhibitory concentration for the visible growth of the tested fungi.

## 4. Conclusions

In summary, this study describes the whole process of separation and structure identification of three novel compounds, koninginins X-Z (**1**–**3**). Their structures including the absolute configurations are clearly characterized. Koninginin X (**1**) and koninginin Y (**2**) share a cyclohexanone concatenate double furan fused-ring system as the critical structure backbone, while koninginin Z (**3**) possesses a bicyclic pyran skeleton. These findings not only greatly elevate the chemical diversity of secondary metabolites for koninginin derivatives, but also further enrich the chemical compositions of the genus of *Trichoderma koningiopsis*.

## Figures and Tables

**Table 1:** ^1^H and ^13^C NMR (*δ*_C_) data for **1**–**3**.

No.	1 ^a^		2 ^a^		3 ^b^	
	*δ*_H_ (*J* in Hz)	*δ*_C_	*δ*_H_ (*J* in Hz)	*δ*_C_	*δ*_H_ (*J* in Hz)	*δ*_C_
1		194.0		194.6		199.4
2	2.42 (1H, m); 2.58 (1H, m)	35.3	2.71 (1H, m); 2.56 (1H, m)	22.5	2.63 (1H, m); 2.29 (1H, m)	34.4
3	2.43 (1H, m); 2.36 (1H, m)	31.1	2.46 (1H, m); 1.90 (1H, m)	29.7	2.18 (1H, m); 1.97 (1H, m)	30.6
4	4.75 (1H, dd, 9.0, 5.4)	64.0	4.14 (1H, d, 4.8)	71.7	4.38 (1H, td, 5.4, 2.4)	67.1
5		178.5		180.6		174.9
6		115.1		111.3		113.2
7	5.25 (1H, dt, 6.0)	79.4	5.65 (1H, d, 6.0)	78.3	4.35 (1H, t, 2.4)	65.4
8	5.25 (1H, dt, 6.0)	89.5	5.05 (1H, d, 6.0)	93.5	2.03 (1H, dt 14.4, 2.4); 1.55 (1H, m)	29.3
9	4.35 (1H, d, 4.2)	72.0	4.16 (1H, d, 4.8)	74.6	4.14 (1H, td, 12.6, 2.4)	78.2
10	3.66 (1H, m)	82.1	3.43 (1H, td, 7.2)	78.6	3.68 (1H, td, 12.6, 6.0)	73.6
11	1.67 (2H, m)	27.7	1.70 (1H, m); 1.64 (1H, m)	27.1	1.64 (2H, m)	33.8
12	1.29 (2H, m)	26.1	1.31 (2H, m)	26.0	1.55 (1H, m); 1.41 (1H, m)	26.8
13	1.29 (2H, m)	29.3	1.31 (2H, m)	29.4	1.41 (1H, m); 1.33 (1H, m)	30.6
14	1.29 (2H, m)	31.7	1.31 (2H, m)	31.6	1.33 (2H, m)	33.2
15	1.29 (2H, m)	22.6	1.31 (2H, m)	22.7	1.33 (2H, m)	23.8
16	0.87 (3H, t, 6.6)	14.0	0.87 (3H, t, 7.2)	14.0	0.91 (3H, t, 6.6)	14.6
17					3.64 (1H, m); 3.55 (1H, m)	65.4
18					1.15 (3H, t, 6.6)	16.0

^a^ Record in CDCl_3_, 600 MHz for ^1^H, and 150 MHz for ^13^C, *δ* in ppm. ^b^ Record in CD_3_OD, 600 MHz for ^1^H, and 150 MHz for ^13^C, *δ* in ppm.

**Table 2:** Calculated ^13^C chemical shifts (CDCl_3_) of structure **1** fitting to the experimental data of compound **1**.

No.	Exptl. *δ*	1	
		1	abs dev ^c^
1	194.0	192.84	1.16
2	35.3	34.53	0.77
3	31.1	29.98	1.12
4	64.0	64.25	0.25
5	178.5	177.16	1.34
6	115.1	119.53	4.43
7	79.4	79.34	0.06
8	89.5	89.96	0.46
9	72.0	74.36	2.36
11	82.1	79.27	2.83
12	27.7	28.77	1.07
13	26.1	25.42	0.68
14	29.3	28.92	0.38
15	31.7	30.87	0.83
16	22.6	22.83	0.23
		MAE ^a^	1.15
		RMS ^b^	1.60
		*P*_mean_	30.45%
		*P*_rel_	100%

^a^ Mean absolute error. ^b^ Root mean square. ^c^ Absolute deviation of calcd. *δ*_C_.

## Data Availability

All the data including X-ray crystallographic data and UV, HRMS, 1D/2D NMR, and CD spectra are available in this publication and the Supplementary Materials.

## Supplementary Materials

The following supporting information can be downloaded at: https://www.mdpi.com/article/10.3390/molecules28237848/s1, Table S1. X-ray crystallographic data and structure refinement for **3**; Figure S1. HRESIMS spectrum of compound **1**; Figure S2. UV spectrum of compound **1**; Figure S3. ^1^H NMR spectrum of compound **1**; Figure S4. ^13^C NMR spectrum of compound **1**; Figure S5. DEPT 135 spectrum of compound **1** recorded in CDCl_3_; Figure S6. ^1^H-^1^H COSY spectrum of compound **1** recorded in CDCl_3_; Figure S7. HSQC spectrum of compound **1** recorded in CDCl_3_; Figure S8. HMBC spectrum of compound **1** recorded in CDCl_3_; Figure S9. NOE difference spectrum of compound **1** recorded in CDCl_3_; Figure S10. CD spectrum of compound **1**; Figure S11. Regression analyses of experimental and calculated ^13^C NMR chemical shifts for **1**; Figure S12. Experimental and calculated ECD spectra of compound **1**; Figure S13. HRESIMS spectrum of compound **2**; Figure S14. UV spectrum of compound **2**; Figure S15. ^1^H NMR spectrum of compound **2**; Figure S16. ^13^C NMR spectrum of compound **2**; Figure S17. DEPT 135 spectrum of compound **2** recorded in CDCl_3_; Figure S18. ^1^H-^1^H COSY spectrum of compound **2** recorded in CDCl_3_; Figure S19. HSQC spectrum of compound **2** recorded in CDCl_3_; Figure S20. HMBC spectrum of compound **2** recorded in CDCl_3_; Figure S21. NOE difference spectrum of compound **2** recorded in CDCl_3_; Figure S22. NOE difference spectrum of compound **2** recorded in CDCl3; Figure S23. NOE difference spectrum of compound **2** recorded in CDCl3; Figure S24. CD spectrum of compound **2**; Figure S25. Experimental and calculated ECD spectra of compound **2**; Figure S26. HRESIMS spectrum of compound **3**; Figure S27. UV spectrum of compound **3**; Figure S28. ^1^H NMR spectrum of compound **3**; Figure S29. ^13^C NMR spectrum of compound **3**; Figure S30. DEPT 135 spectrum of compound **3** recorded in CD_3_OD; Figure S31. ^1^H-^1^H COSY spectrum of compound **3** recorded in CD_3_OD; Figure S32. HSQC spectrum of compound **3** recorded in CD_3_OD; Figure S33. HMBC spectrum of compound **3** recorded in CD_3_OD; Figure S34. NOESY spectrum of compound **3** recorded in CD_3_OD; Figure S35. CD spectrum of compound **3**; Figure S36. Experimental and calculated ECD spectra of compound **3**.
