# Supplementary material for: Koninginins X-Z, Three New Polyketides from Trichoderma koningiopsis SC-5
Source: Molecules. 2023 Nov 29;28(23):7848. doi: 10.3390/molecules28237848 (PMC10707852; doi:10.3390/molecules28237848)
Supplement: Supplementary file 1 [file molecules-28-07848-s001.zip › molecules-2714937-supplementary.pdf]

SUPPLEMENTARY MATERIAL

Koninginins X-Z, three new polyketides from *Trichoderma koningiopsis* SC-5

Weiwei Peng [1,2,†], Jianbing Tan [1,2,†], Zihuan Sang [1,2,3], Yuantao Huang [4], Li Xu [1,2], Yuting Zheng [1,2], Siyu Qin [1,2], Haibo Tan [1,2,3,*], Zhenxing Zou [1,2,*]

[1] Xiangya School of Pharmaceutical Sciences, Central South University, Changsha, China; pww199802@163.com (W.P.); tanjb1009@csu.edu.cn (J.T.); sangzihuan123@163.com (Z.S.); 217211012@csu.edu.cn (L.X.); 217211011@csu.edu.cn (Y.Z.); siyuqin1226@163.com (S.Q.).

[2] Hunan Key Laboratory of Diagnostic and Therapeutic Drug Research for Chronic Diseases, Changsha, China.

[3] Key Laboratory of South China Agricultural Plant Molecular Analysis and Genetic Improvement, Guangdong Provincial Key Laboratory of Applied Botany, South China Botanical Garden, Chinese Academy of Sciences, Guangzhou, China

[4] Affiliated Haikou Hospital of Xiangya School of Medicine, Central South University, Haikou, China
hyt951232@163.com (Y.H.);

* Correspondence: tanhaibo@scbg.ac.cn (H.T.); zouzhenxing@csu.edu.cn (Z.Z.); Tel.: +86-731-82650395 (Z.Z.)

† These authors contributed equally to this work.

List of Contents..Page

Table S1. X-ray crystallographic data and structure refinement for **3**............3
Figure S1. HRESIMS spectrum of compound **1**..4
Figure S2. UV spectrum of compound **1**...4
Figure S3. ^{1}H NMR spectrum (600 MHz, $CDCl_3$) of compound **1**................5
Figure S4. ^{13}C NMR spectrum (150 MHz, $CDCl_3$) of compound **1**................5
Figure S5. DEPT 135 spectrum of compound **1** recorded in $CDCl_3$...............6
Figure S6. ^{1}H-^{1}H COSY spectrum of compound **1** recorded in $CDCl_3$...........6
Figure S7. HSQC spectrum of compound **1** recorded in $CDCl_3$.....................7
Figure S8. HMBC spectrum of compound **1** recorded in $CDCl_3$...................7
Figure S9. NOE difference spectrum of compound **1** recorded in $CDCl_3$.......8
Figure S10. CD spectrum of compound **1**..8
Figure S11. Regression analyses of experimental and calculated ^{13}C NMR chemical shifts for **1**...9
Figure S12. Experimental and calculated ECD spectra of compound **1**..........9
Figure S13. HRESIMS spectrum of compound **2**..10
Figure S14. UV spectrum of compound **2**...10
Figure S15. ^{1}H NMR spectrum (600 MHz, $CDCl_3$) of compound **2**............11
Figure S16. ^{13}C NMR spectrum (150 MHz, $CDCl_3$) of compound **2**...........11
Figure S17. DEPT 135 spectrum of compound **2** recorded in $CDCl_3$..........12
Figure S18. ^{1}H-^{1}H COSY spectrum of compound **2** recorded in $CDCl_3$.......12
Figure S19. HSQC spectrum of compound **2** recorded in $CDCl_3$.................13
Figure S20. HMBC spectrum of compound **2** recorded in $CDCl_3$...............13
Figure S21. NOE difference spectrum of compound **2** recorded in $CDCl_3$...14
Figure S22. NOE difference spectrum of compound **2** recorded in $CDCl_3$...14
Figure S23. NOE difference spectrum of compound **2** recorded in $CDCl_3$...15
Figure S24. CD spectrum of compound **2**..15
Figure S25. Experimental and calculated ECD spectra of compound **2**........16
Figure S26. HRESIMS spectrum of compound **3**..16
Figure S27. UV spectrum of compound **3**...17
Figure S28. ^{1}H NMR spectrum (600 MHz, CD_3OD) of compound **3**...........17
Figure S29. ^{13}C NMR spectrum (150 MHz, CD_3OD) of compound **3**..........18
Figure S30. DEPT 135 spectrum of compound **3** recorded in CD_3OD.........18
Figure S31. ^{1}H-^{1}H COSY spectrum of compound **3** recorded in CD_3OD.....19
Figure S32. HSQC spectrum of compound **3** recorded in CD_3OD................19
Figure S33. HMBC spectrum of compound **3** recorded in CD_3OD..............20
Figure S34. NOESY spectrum of compound **3** recorded in CD_3OD............20
Figure S35. CD spectrum of compound **3**...21
Figure S36. Experimental and calculated ECD spectra of compound **3**........21

Table S1. X-ray crystallographic data and structure refinement for **3**

Identification code	**3**
Empirical formula	$C_{18}H_{30}O_5$
Formula weight	326.43
Temperature/K	100.00(10)
Crystal system	trigonal
Space group	$P2_1$
a/Å	5.0609(2)
b/Å	39.7106(9)
c/Å	9.8073(3)
α/°	90
β/°	104.855(3)
γ/°	90
Volume/Å^3	1905.11(11)
Z	2
ρ_{calc}g/cm^3	1.232
μ/mm^1	0.759
F(000)	772.0
Crystal size/mm^3	0.15 × 0.04 × 0.03
Radiation	CuKα (λ = 1.54184)
2Θ range for data collection/°	8.908 to 148.692
Index ranges	$-6 \leqslant h \leqslant 6, -49 \leqslant k \leqslant 49, -12 \leqslant 1 \leqslant 12$
Reflections collected	6178
Independent reflections	6178 [R_{int} = 0.0298, R_{sigma} = 0.0394]
Data/restraints/parameters	6178/1/457
Goodness-of-fit on F^2	1.073
Final R indexes [I>=2σ (I)]	R_1 = 0.0423, ωR_2 = 0.1107
Final R indexes [all data]	R_1 = 0.0454, ωR_2 = 0.1121
Largest diff. peak/hole / e Å^{-3}	0.37/-0.21
Flack parameter	0.15(11)

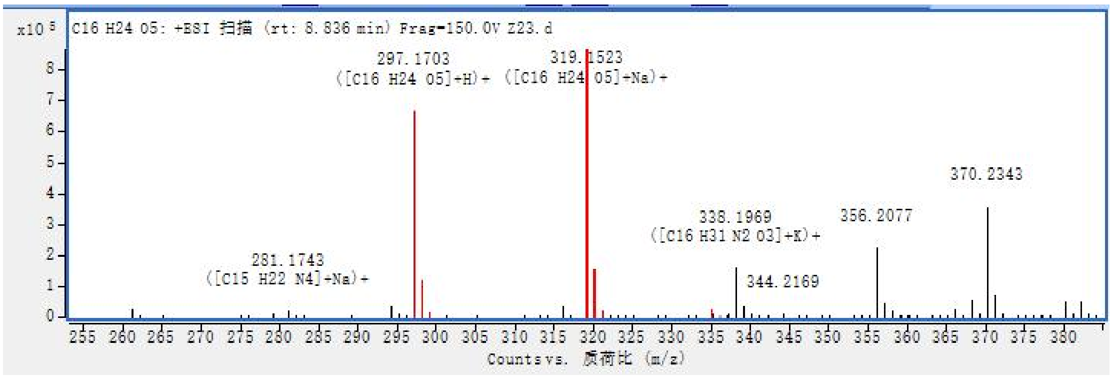

Figure S1. HRESIMS spectrum of compound **1**

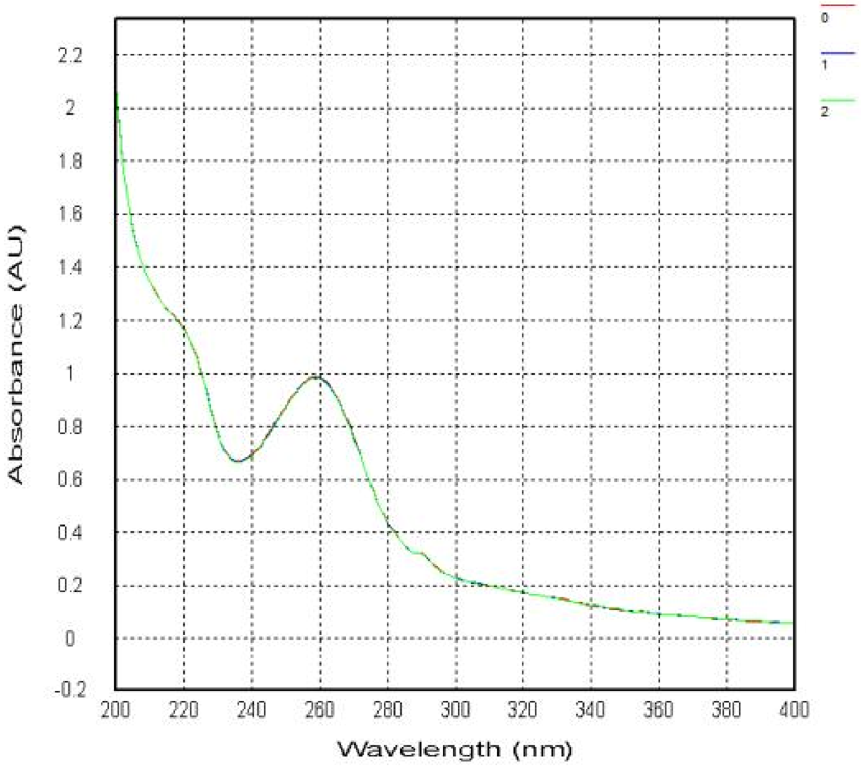

Figure S2. UV spectrum of compound **1**

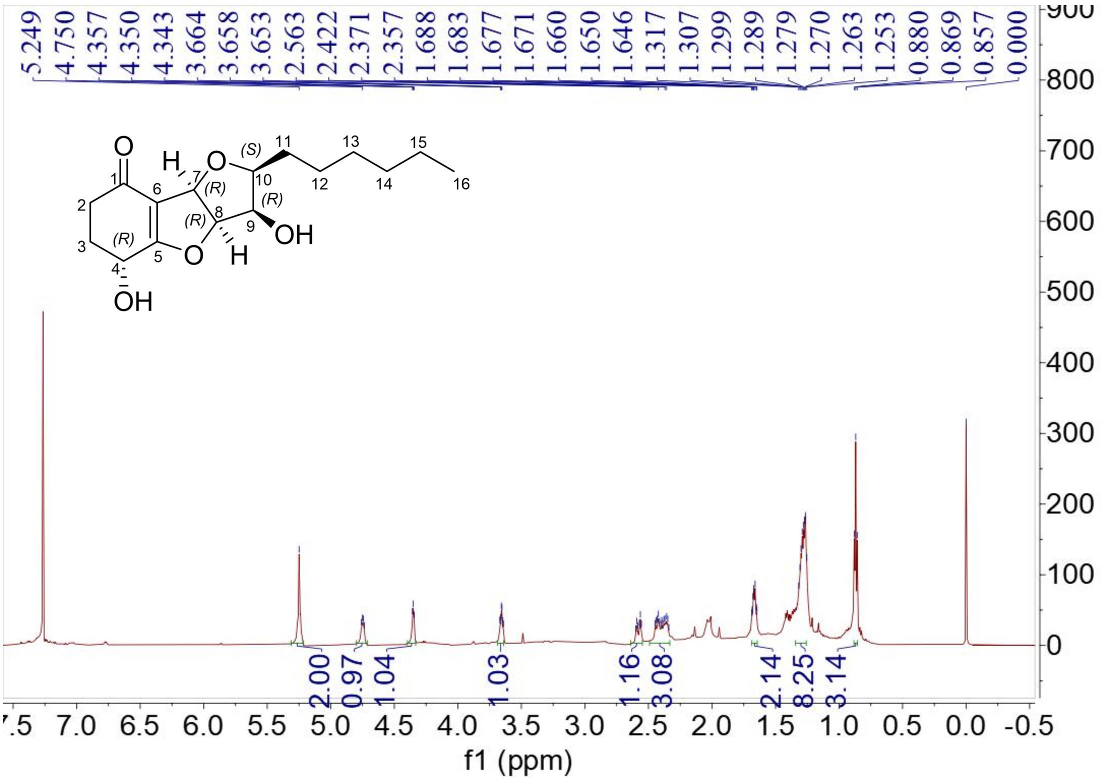

Figure S3. ^{1}H NMR spectrum (600 MHz, $CDCl_3$) of compound **1**

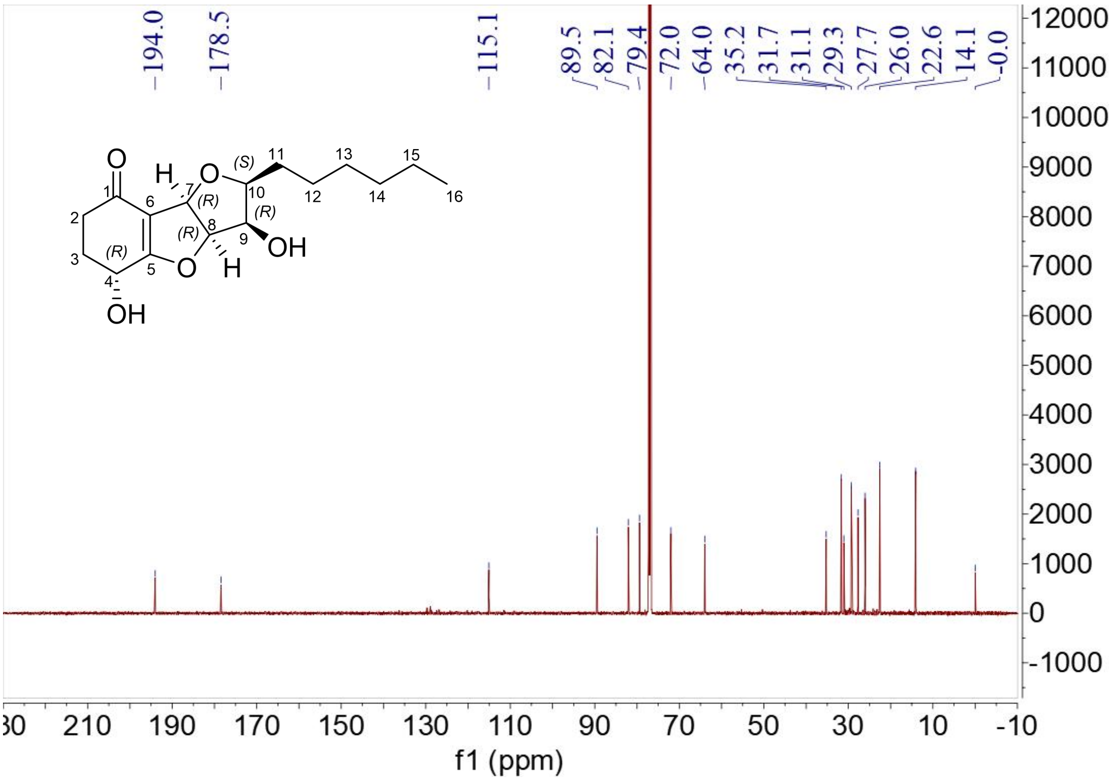

Figure S4. ^{13}C NMR spectrum (150 MHz, $CDCl_3$) of compound **1**

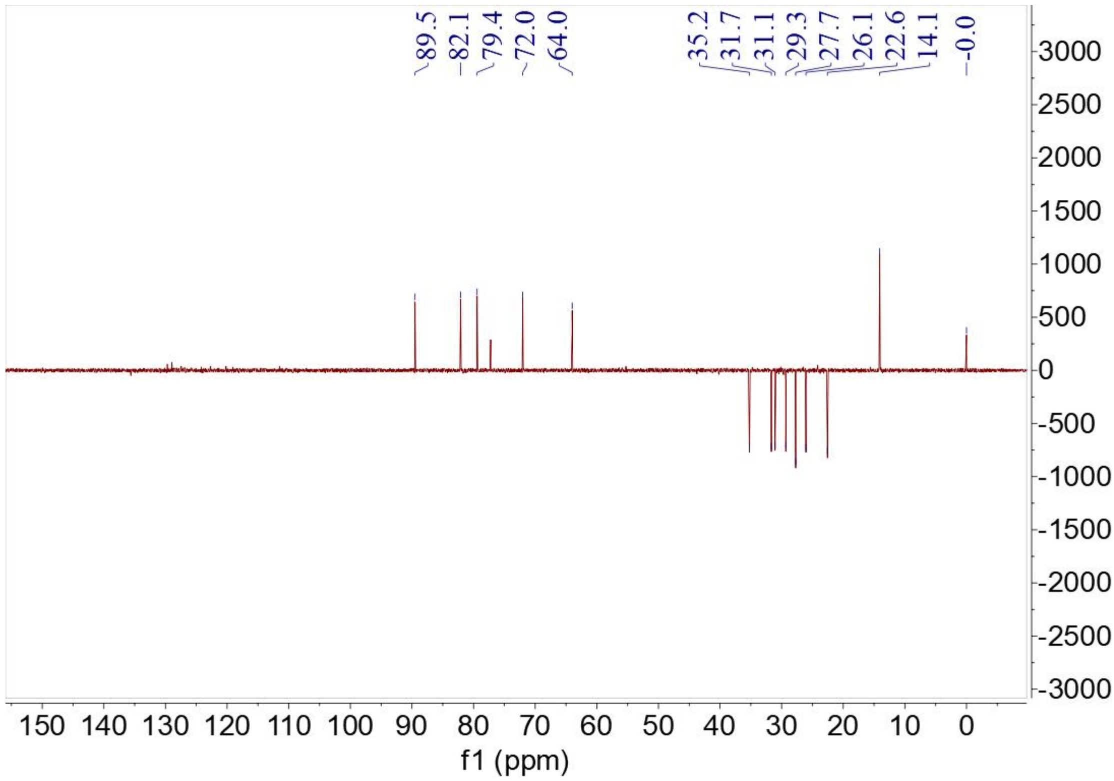

Figure S5. DEPT 135 spectrum of compound **1** recorded in $CDCl_3$

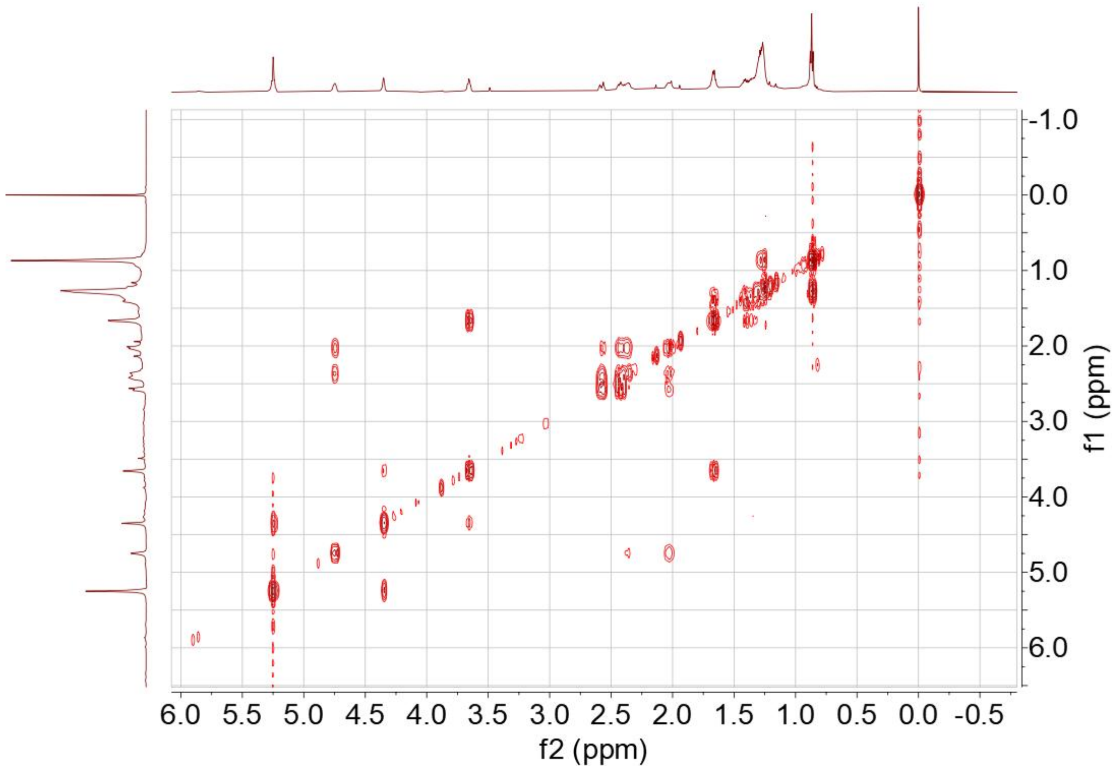

Figure S6. ^1H-^1H COSY spectrum of compound **1** recorded in $CDCl_3$

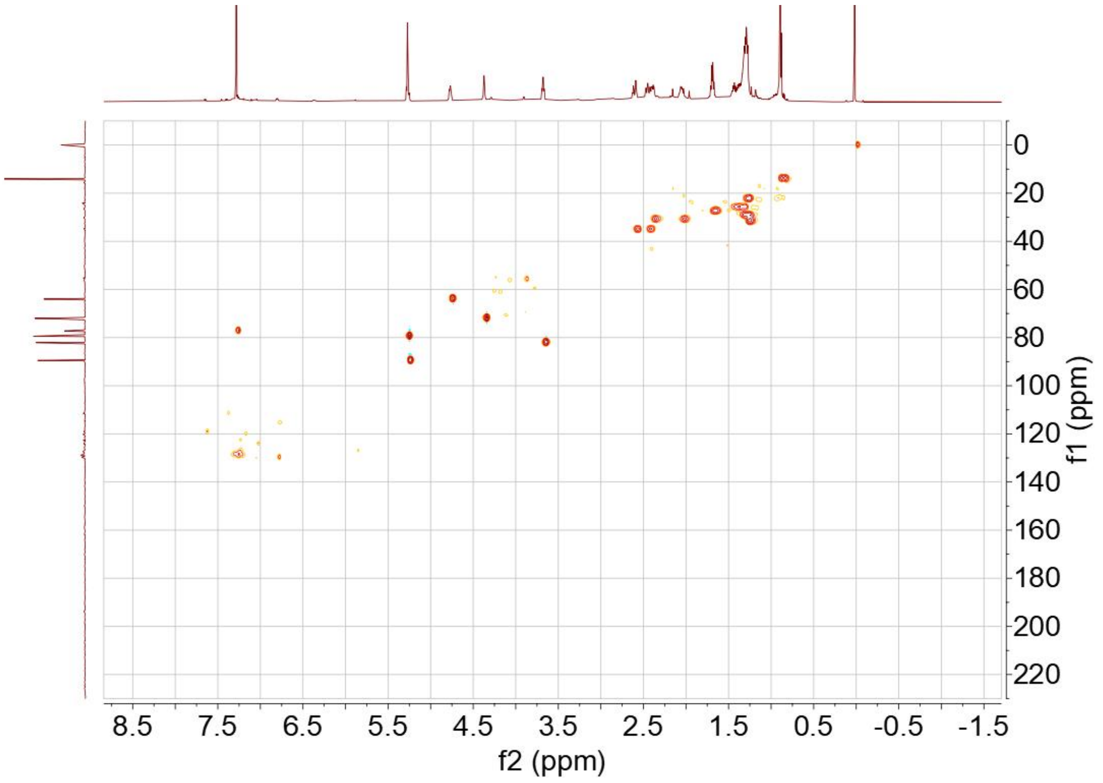

Figure S7. HSQC spectrum of compound **1** recorded in $CDCl_3$

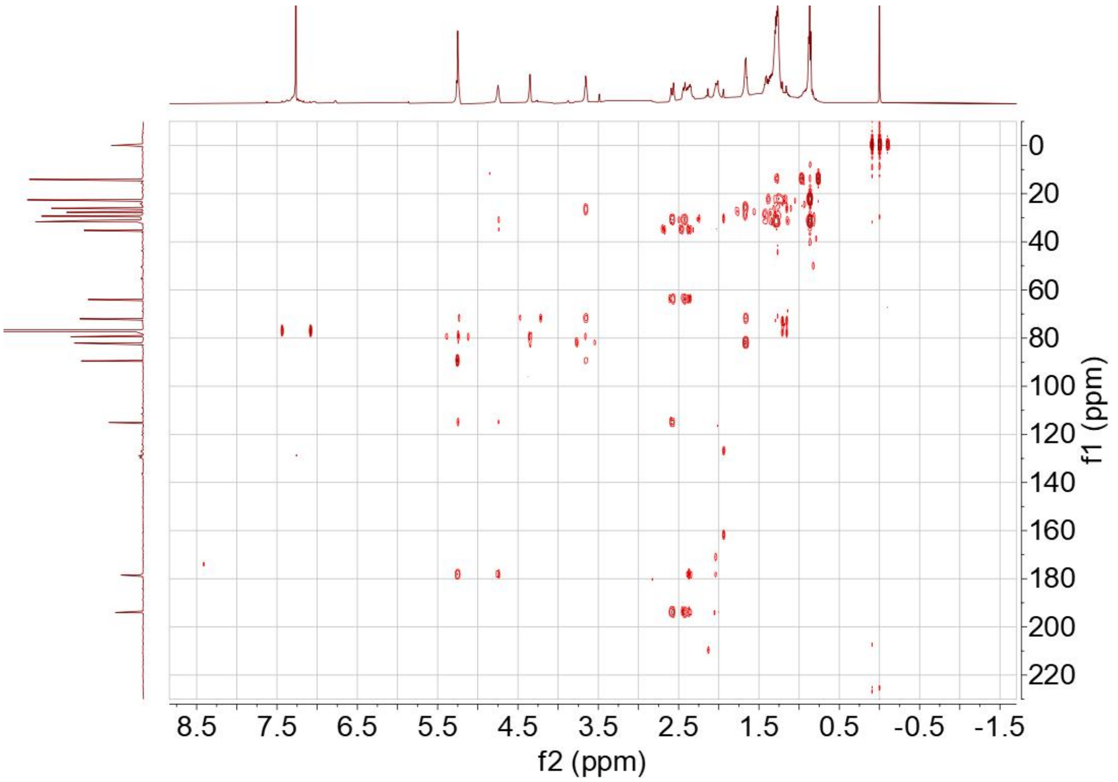

Figure S8. HMBC spectrum of compound **1** recorded in $CDCl_3$

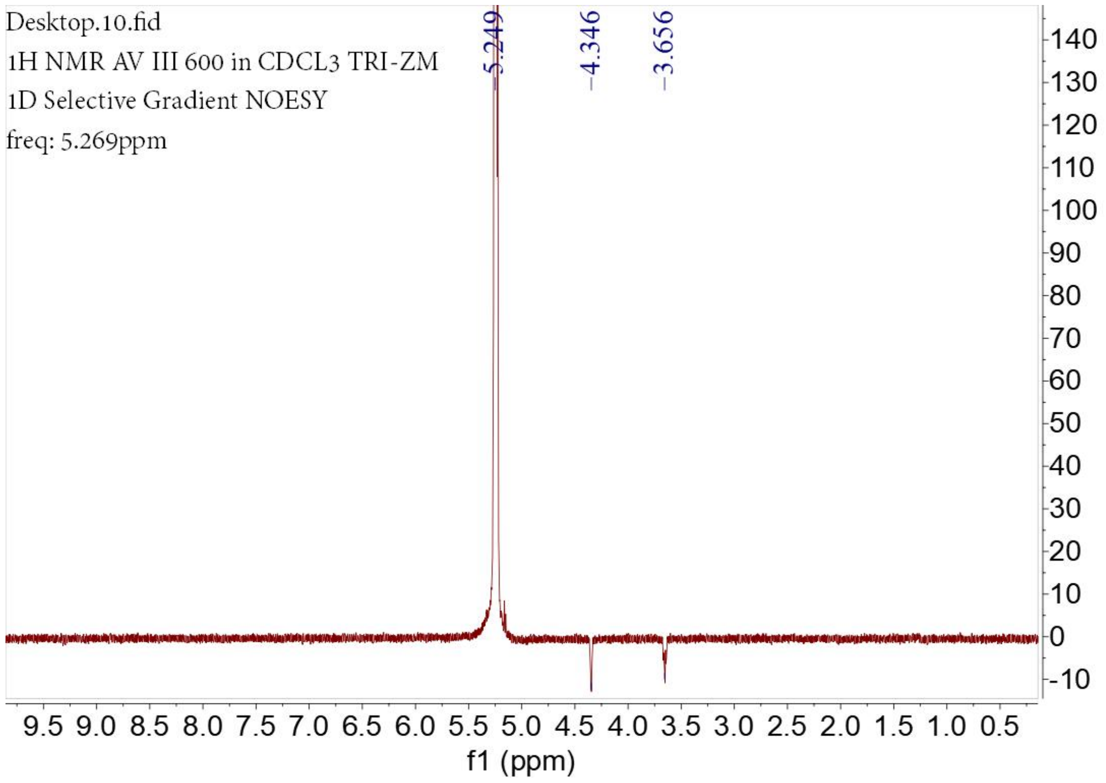

Figure S9. NOE difference spectrum of compound **1** recorded in $CDCl_3$

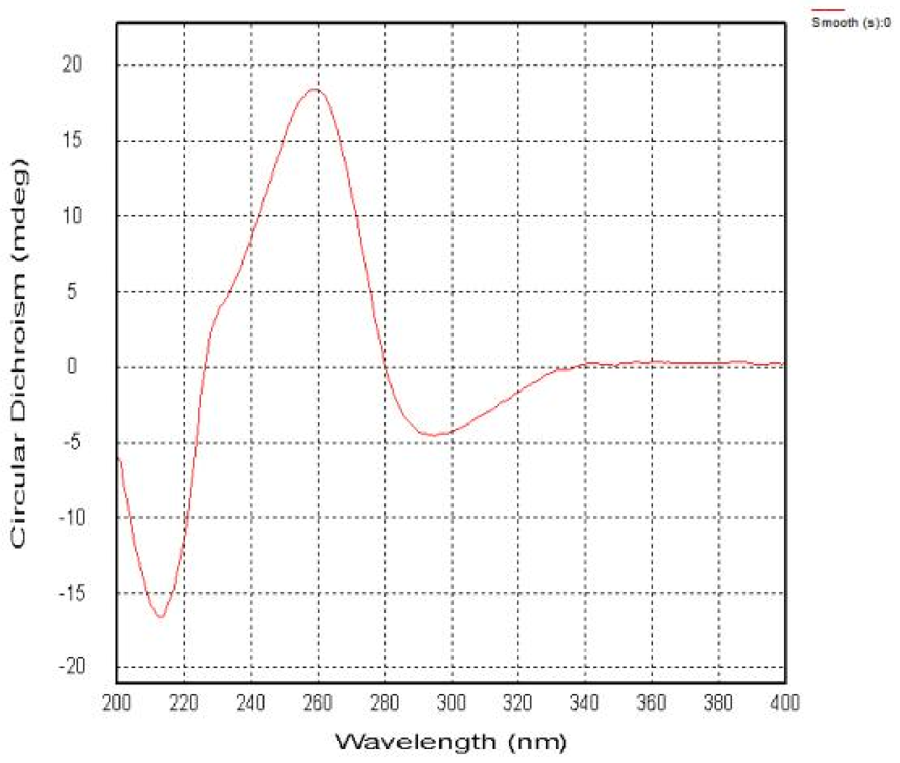

Figure S10. CD spectrum of compound **1**

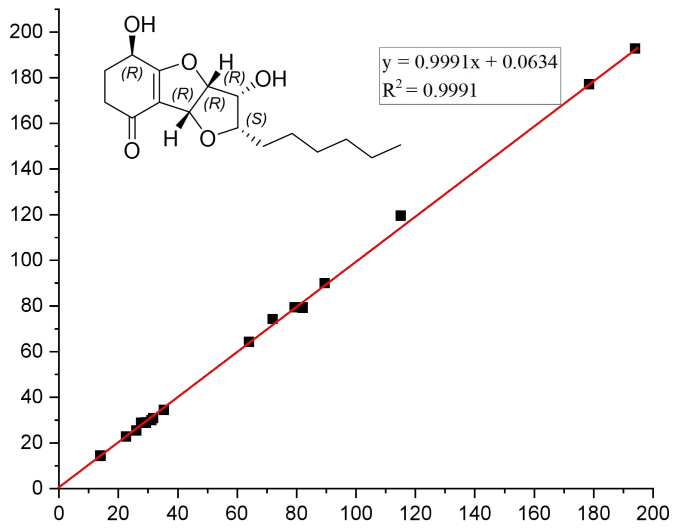

Figure S11. Regression analyses of experimental and calculated ^{13}C NMR chemical shifts for **1**

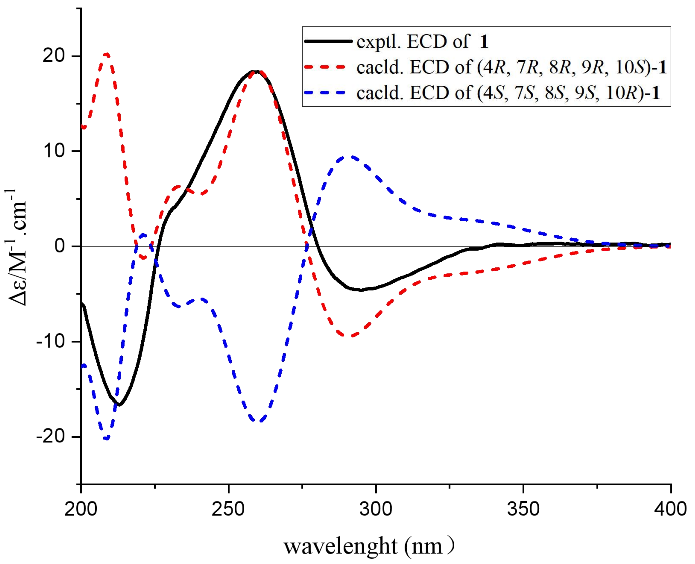

Figure S12. Experimental and calculated ECD spectra of compound **1**

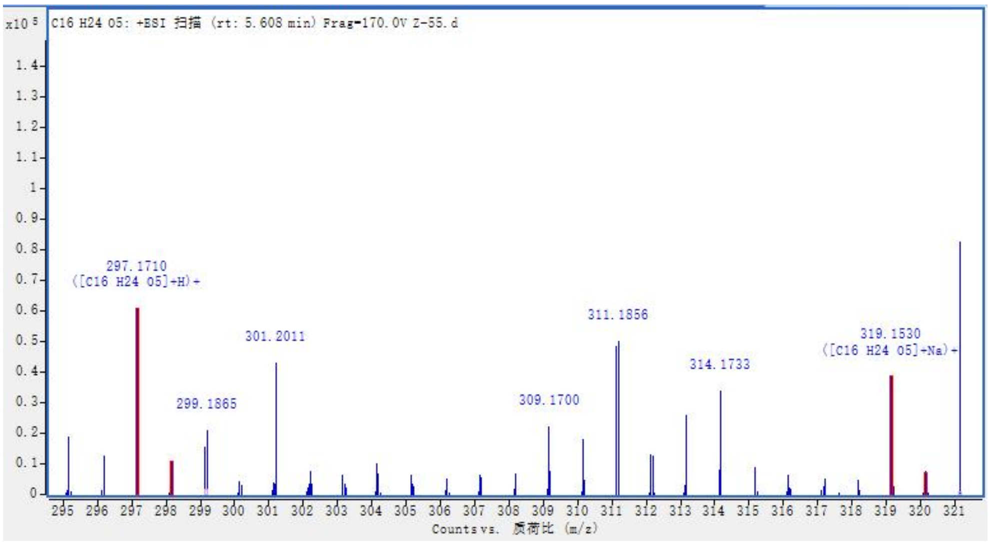

Figure S13. HRESIMS spectrum of compound **2**

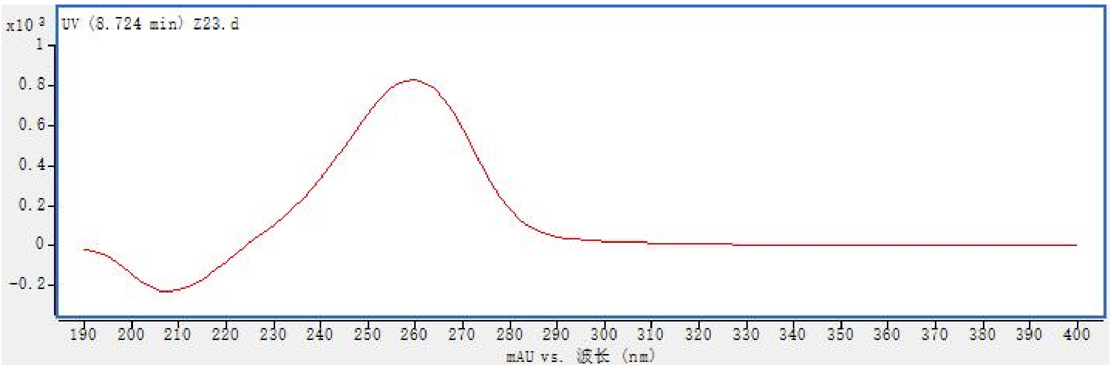

Figure S14. UV spectrum of compound **2**

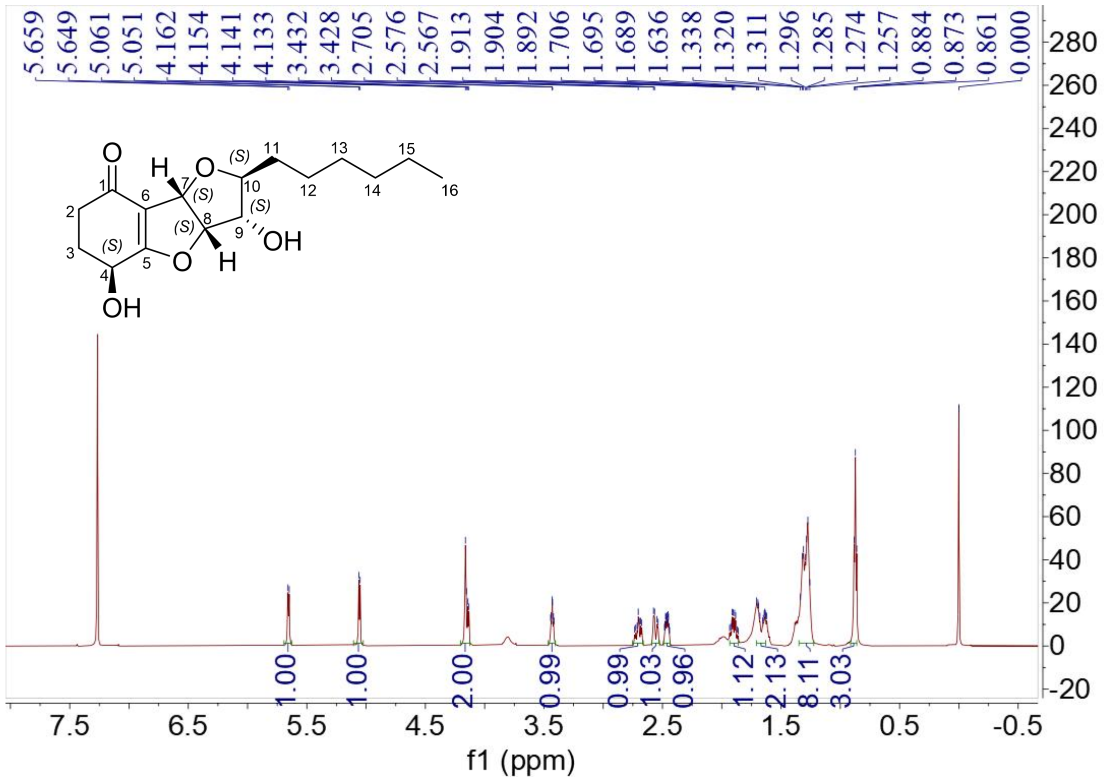

Figure S15. ^{1}H NMR spectrum (600 MHz, $CDCl_3$) of compound **2**

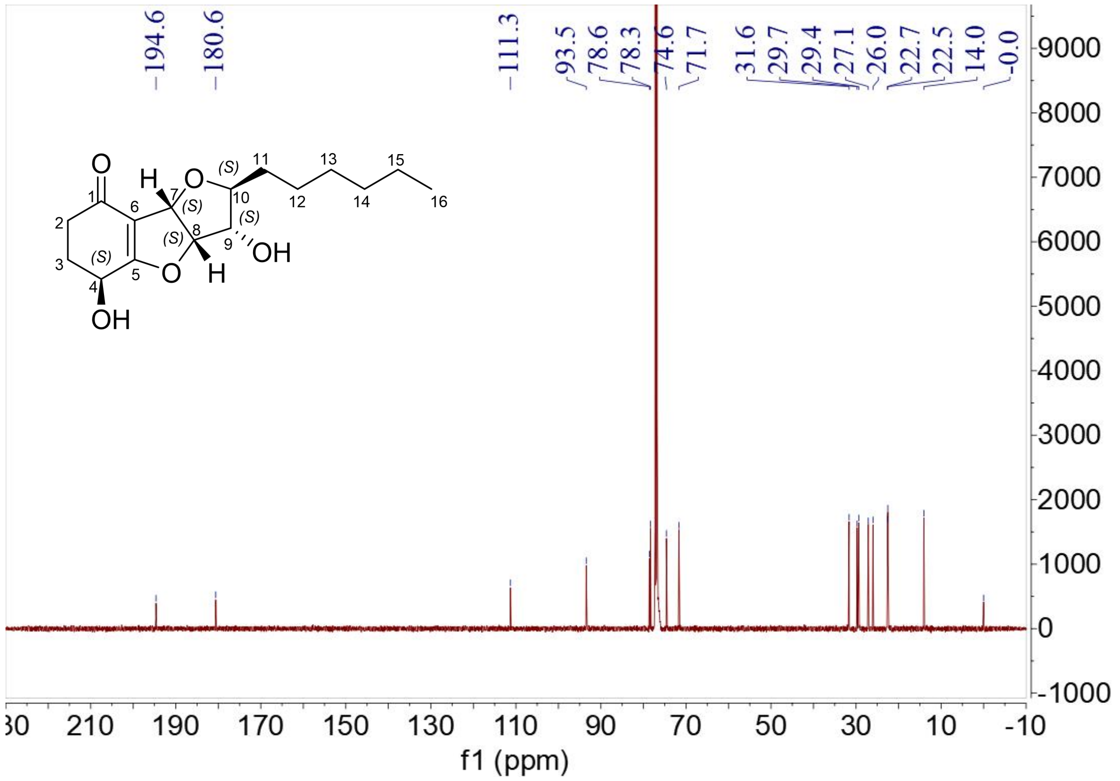

Figure S16. ^{13}C NMR spectrum (150 MHz, $CDCl_3$) of compound **2**

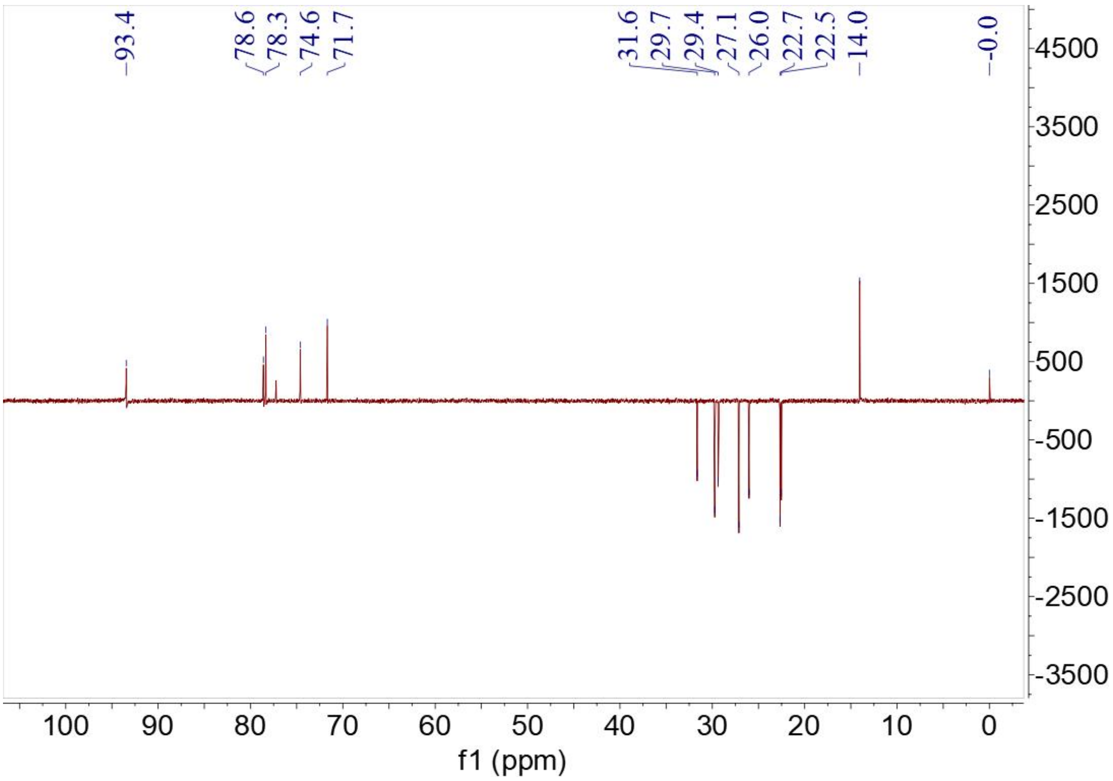

Figure S17. DEPT 135 spectrum of compound **2** recorded in $CDCl_3$

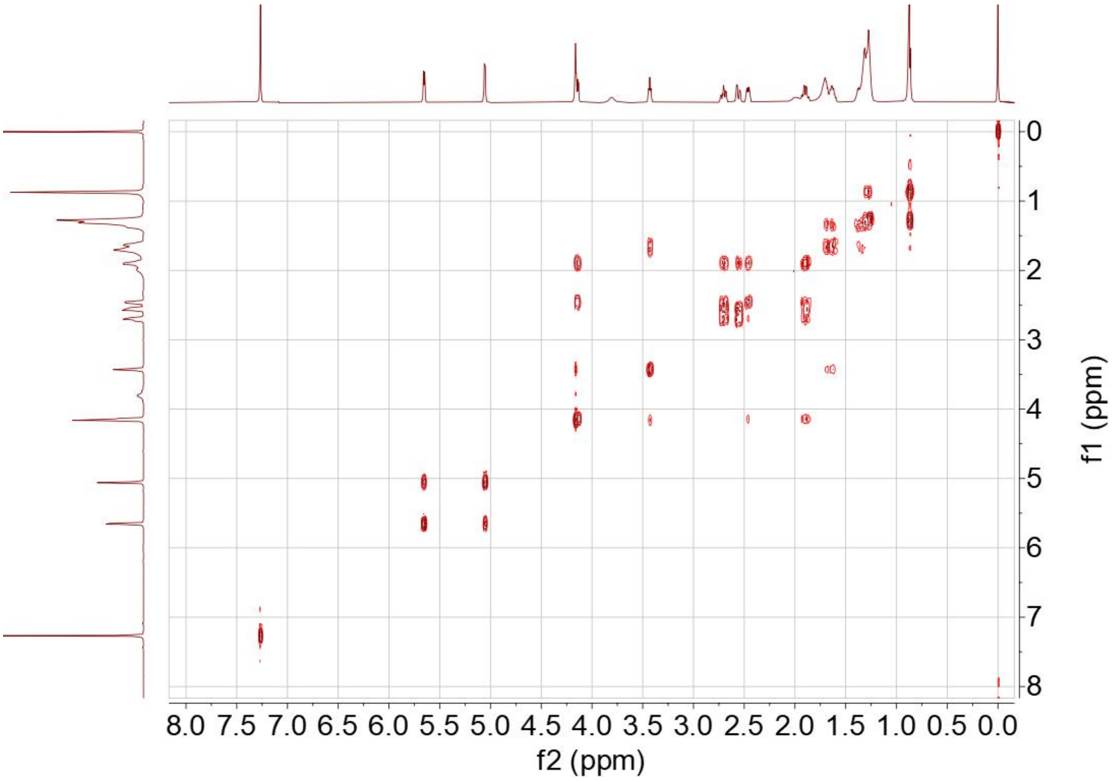

Figure S18. ^{1}H-^{1}H COSY spectrum of compound **2** recorded in $CDCl_3$

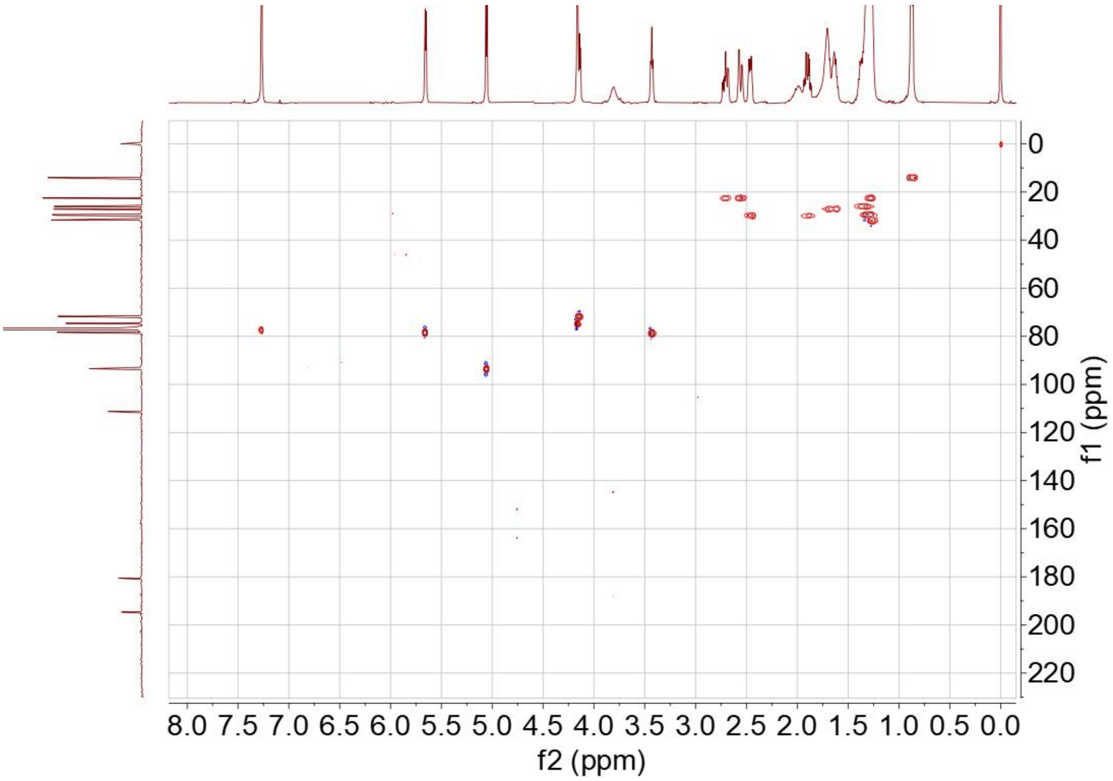

Figure S19. HSQC spectrum of compound **2** recorded in $CDCl_3$

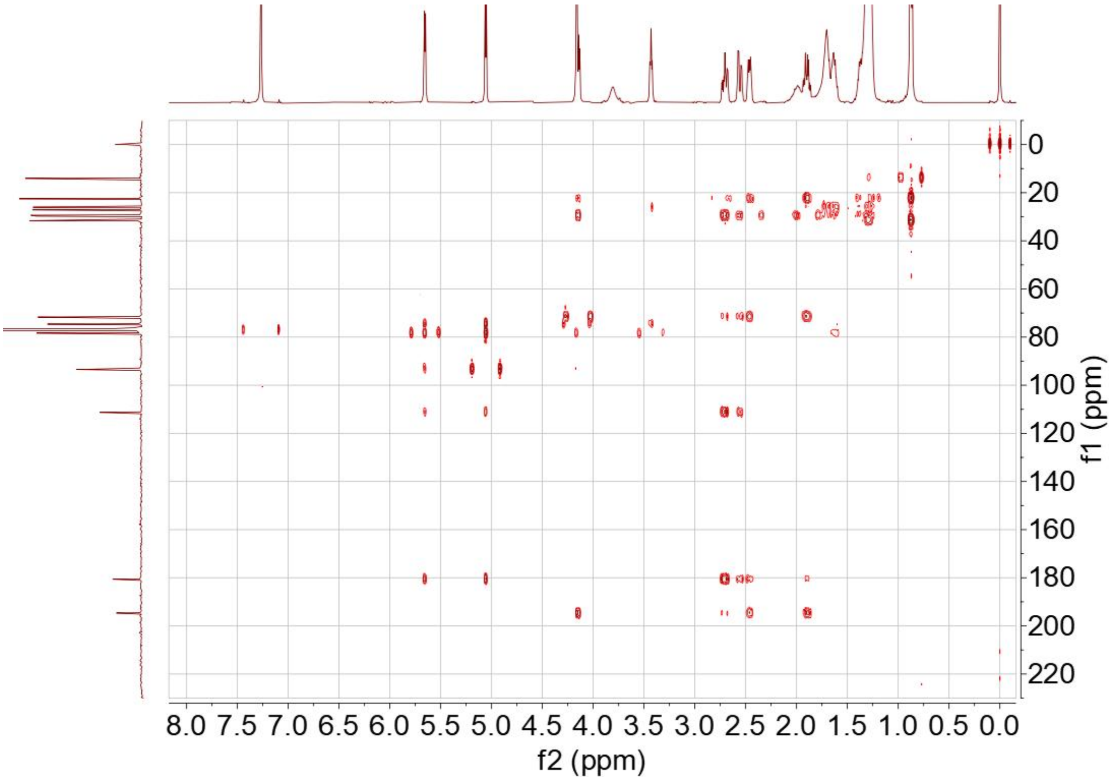

Figure S20. HMBC spectrum of compound **2** recorded in $CDCl_3$

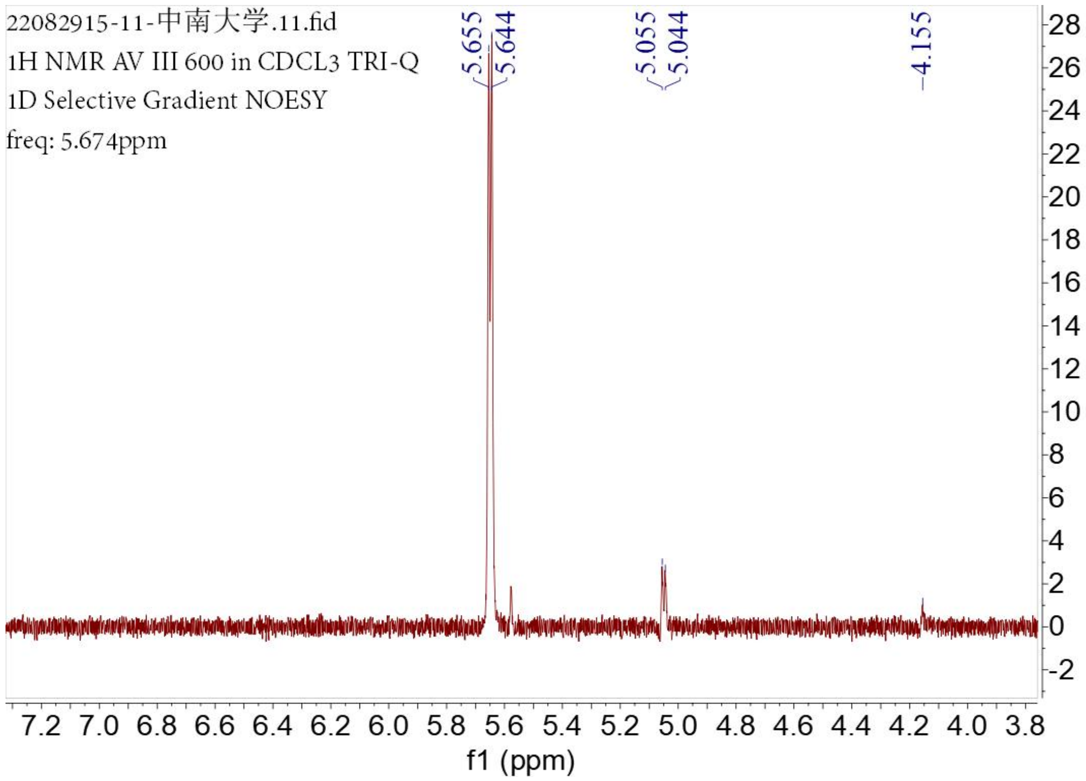

Figure S21. NOE difference spectrum of compound **2** recorded in $CDCl_3$

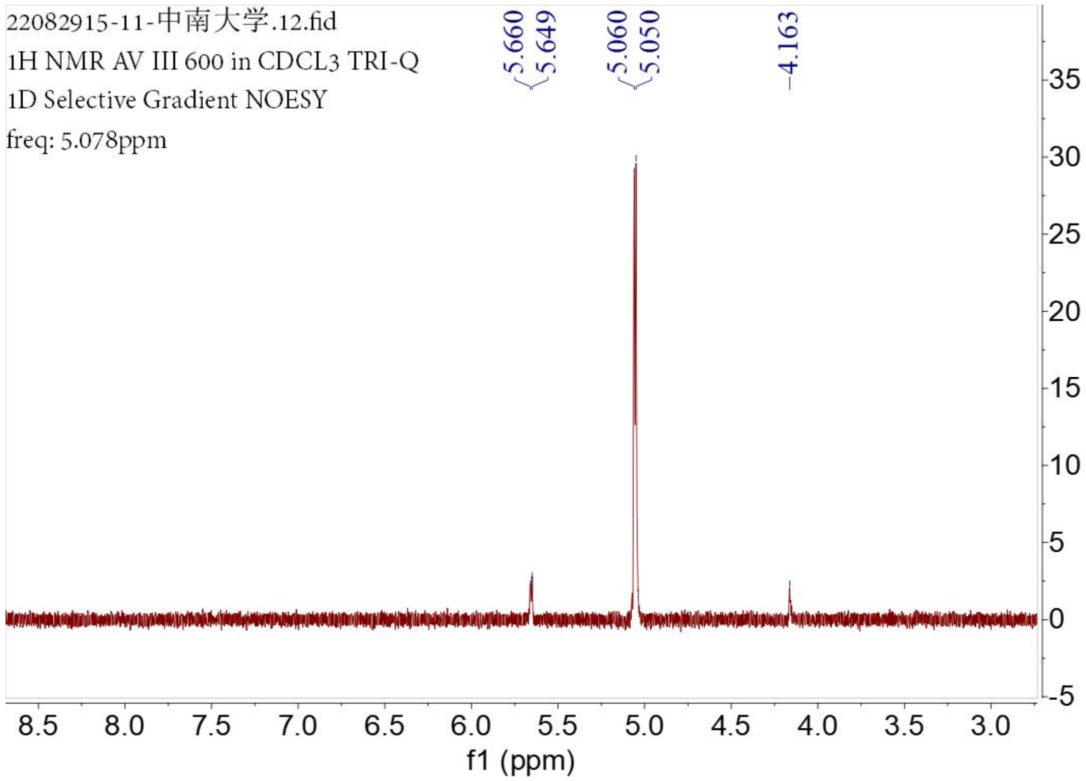

Figure S22. NOE difference spectrum of compound **2** recorded in $CDCl_3$

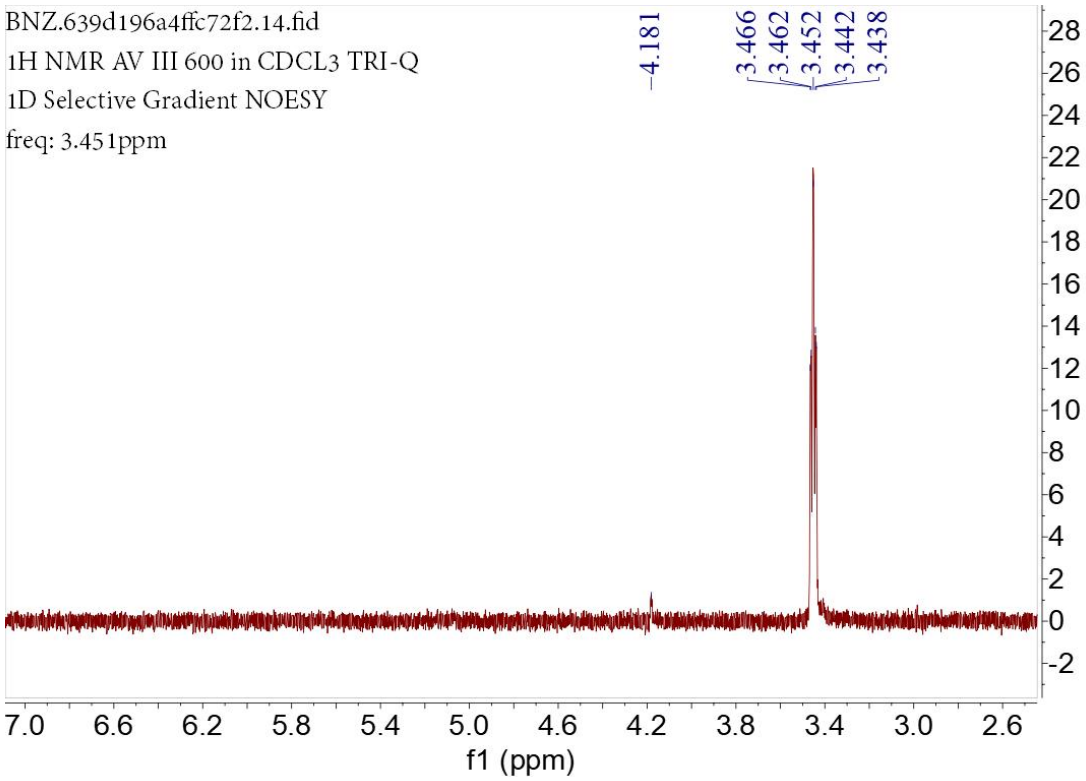

Figure S23. NOE difference spectrum of compound **2** recorded in $CDCl_3$

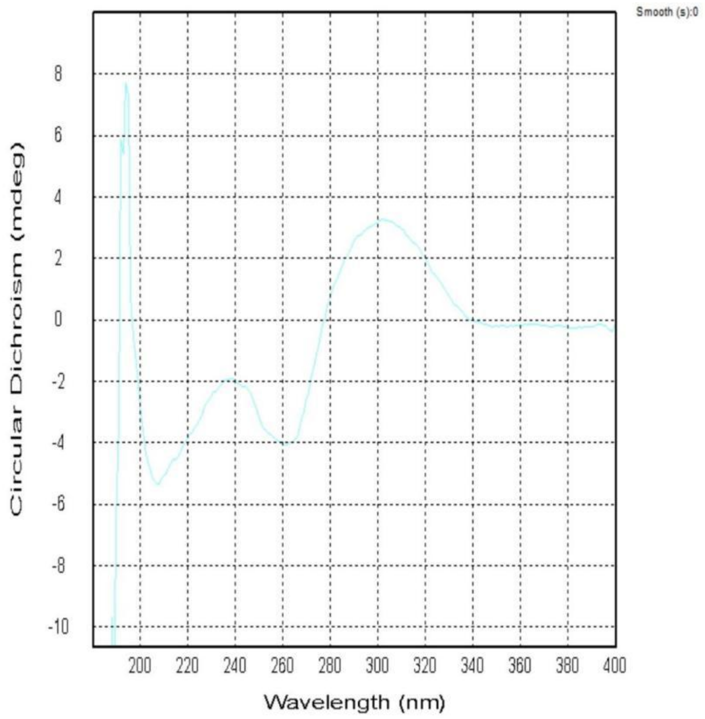

Figure S24. CD spectrum of compound **2**

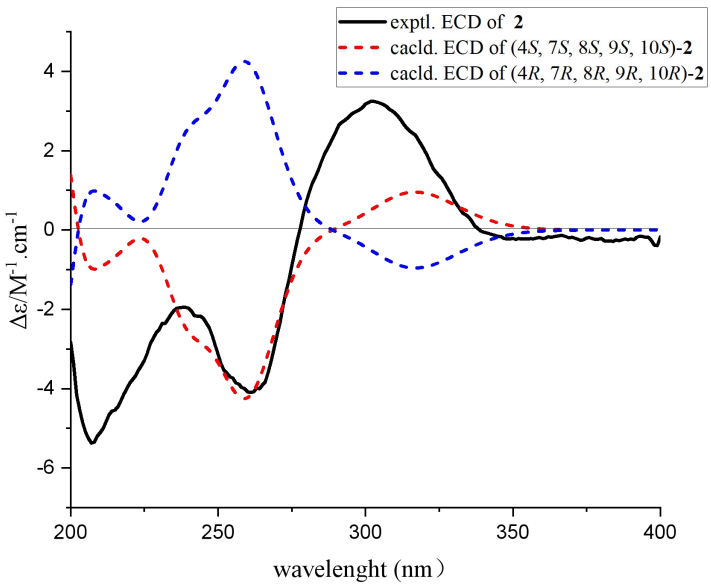

Figure S25. Experimental and calculated ECD spectra of compound **2**

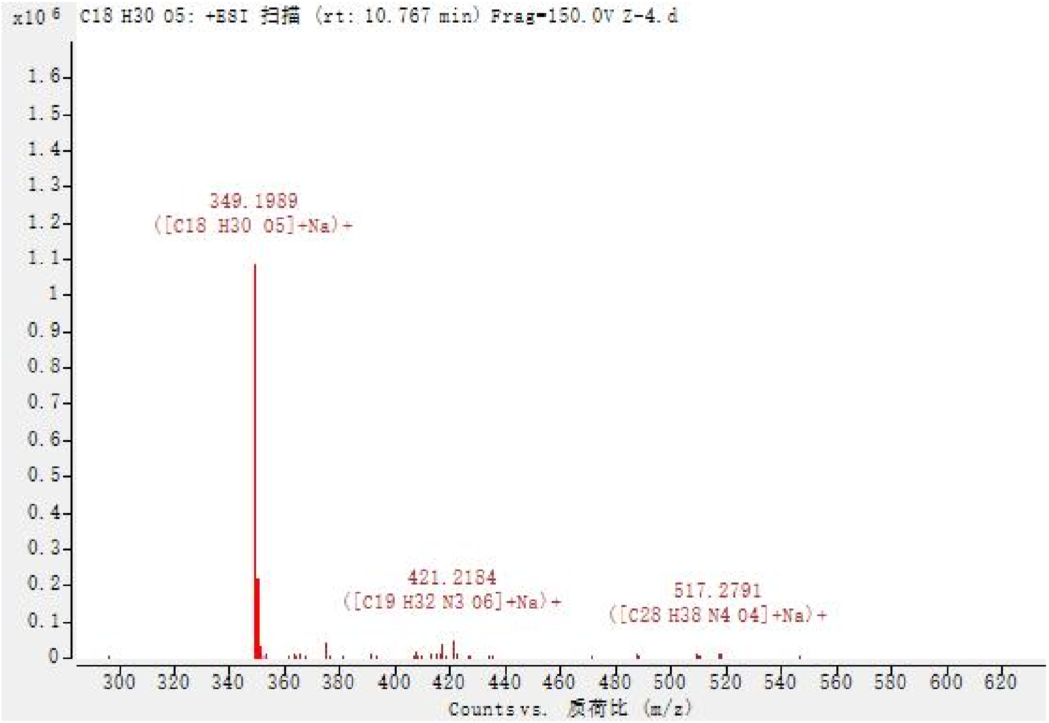

Figure S26. HRESIMS spectrum of compound **3**

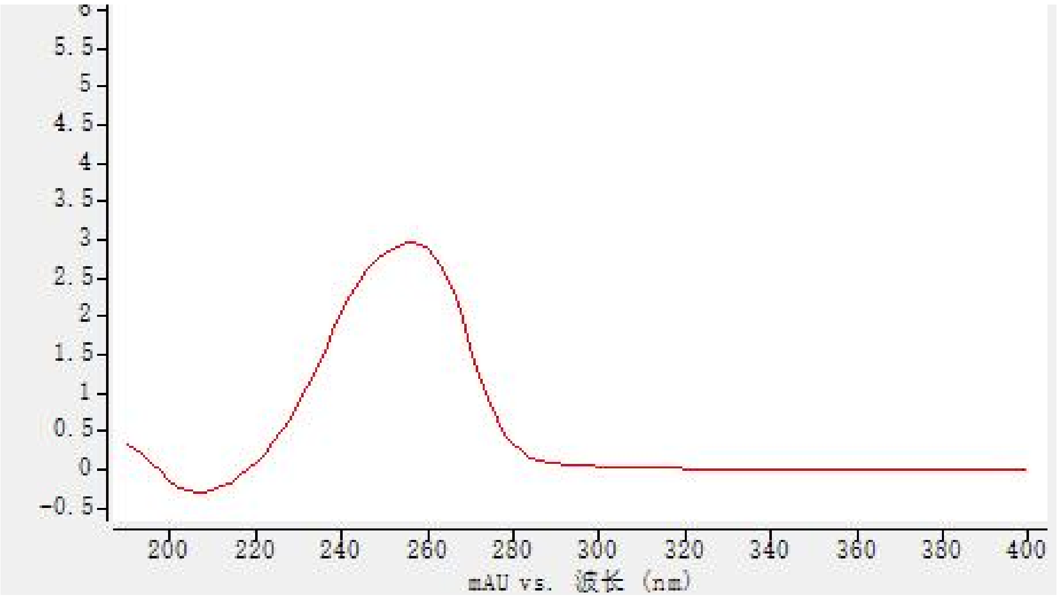

Figure S27. UV spectrum of compound **3**

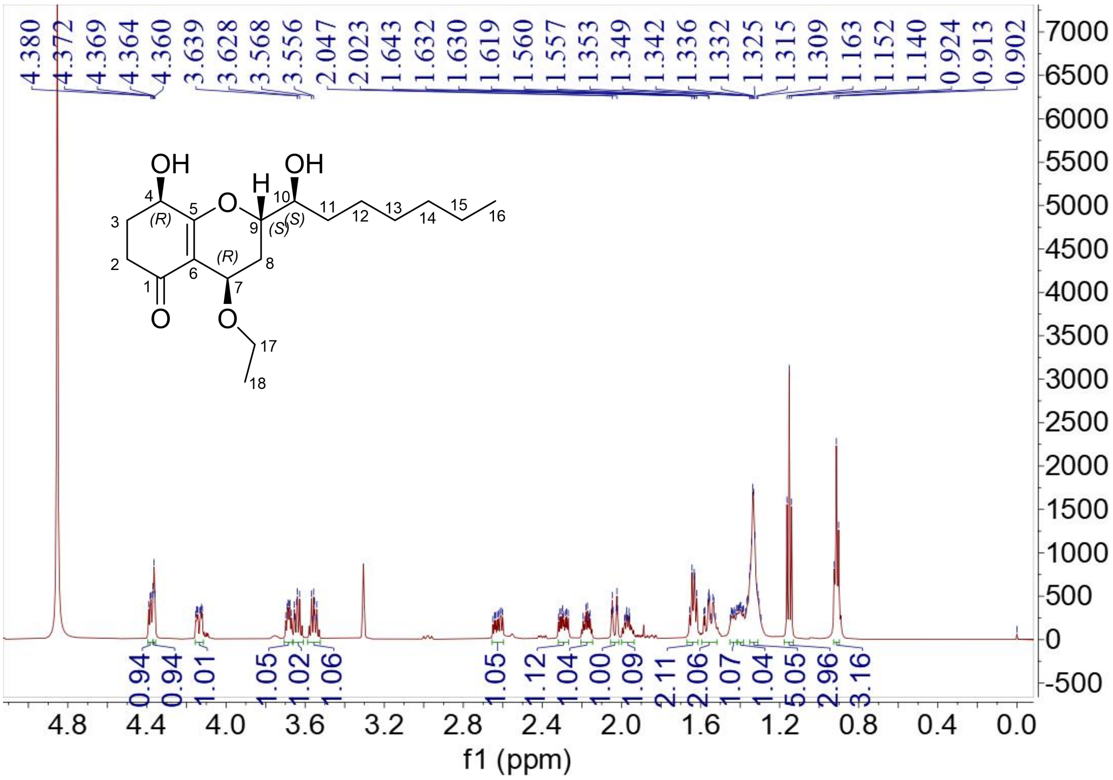

Figure S28. ^{1}H NMR spectrum (600 MHz, CD_3OD) of compound **3**

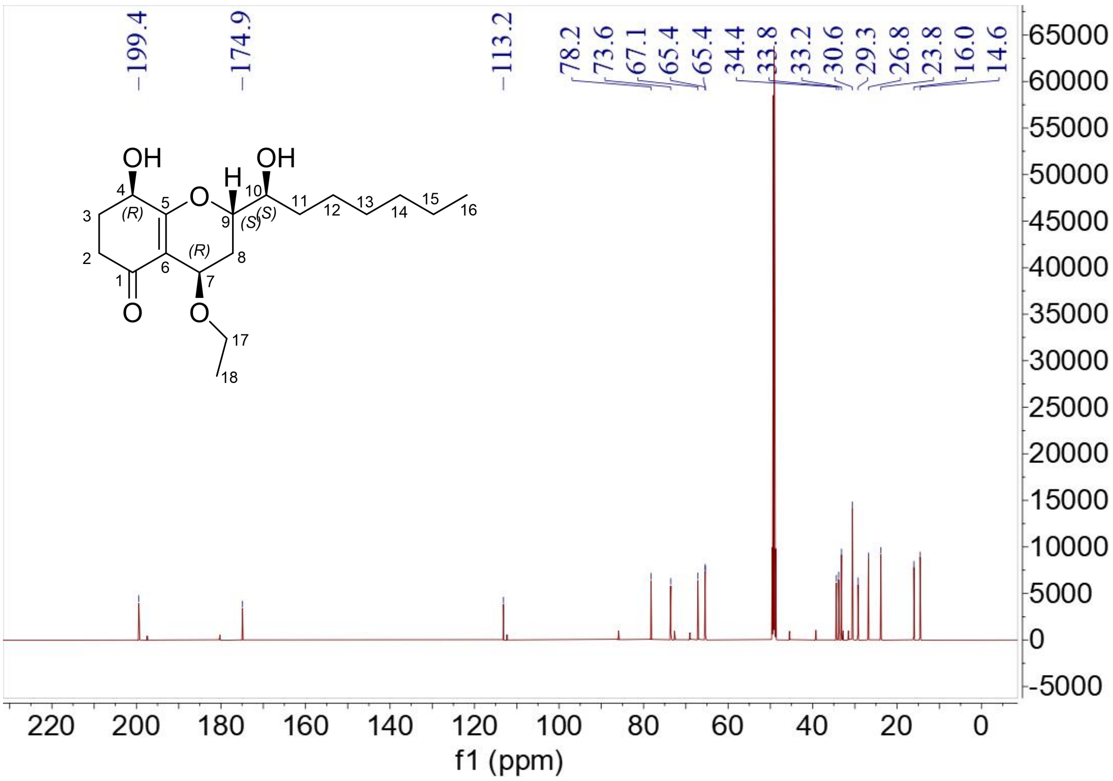

Figure S29. ^{13}C NMR spectrum (150 MHz, CD_3OD) of compound **3**

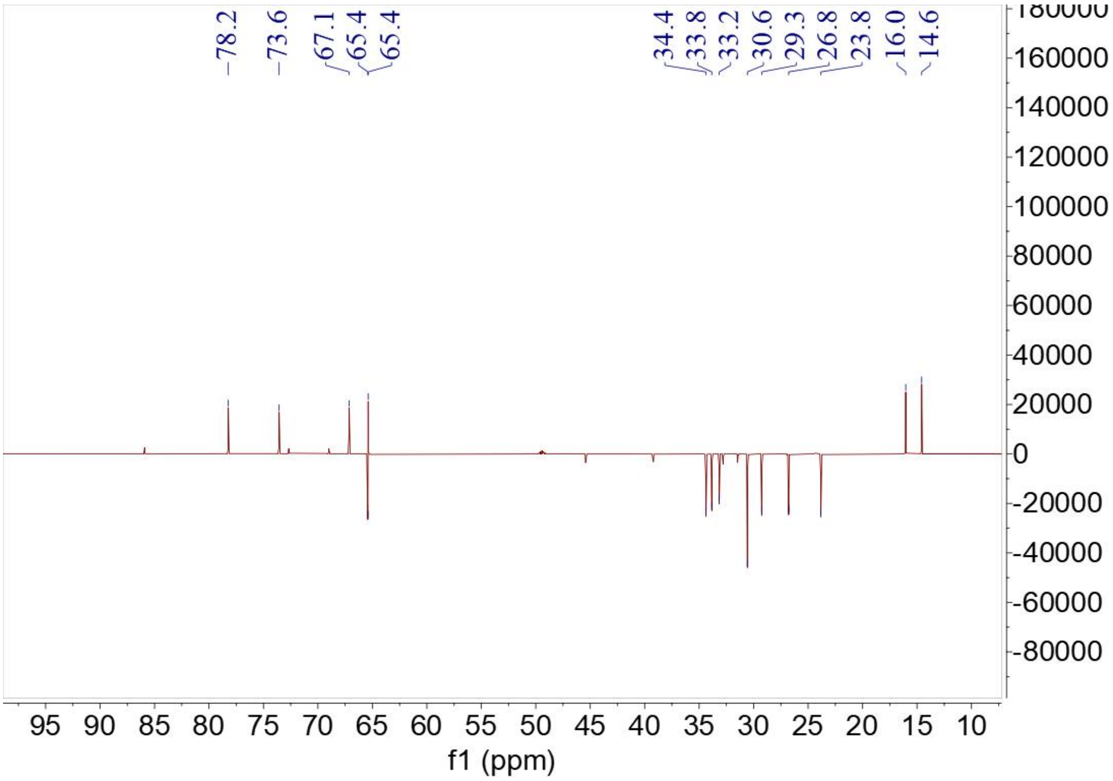

Figure S30. DEPT 135 spectrum of compound **3** recorded in CD_3OD

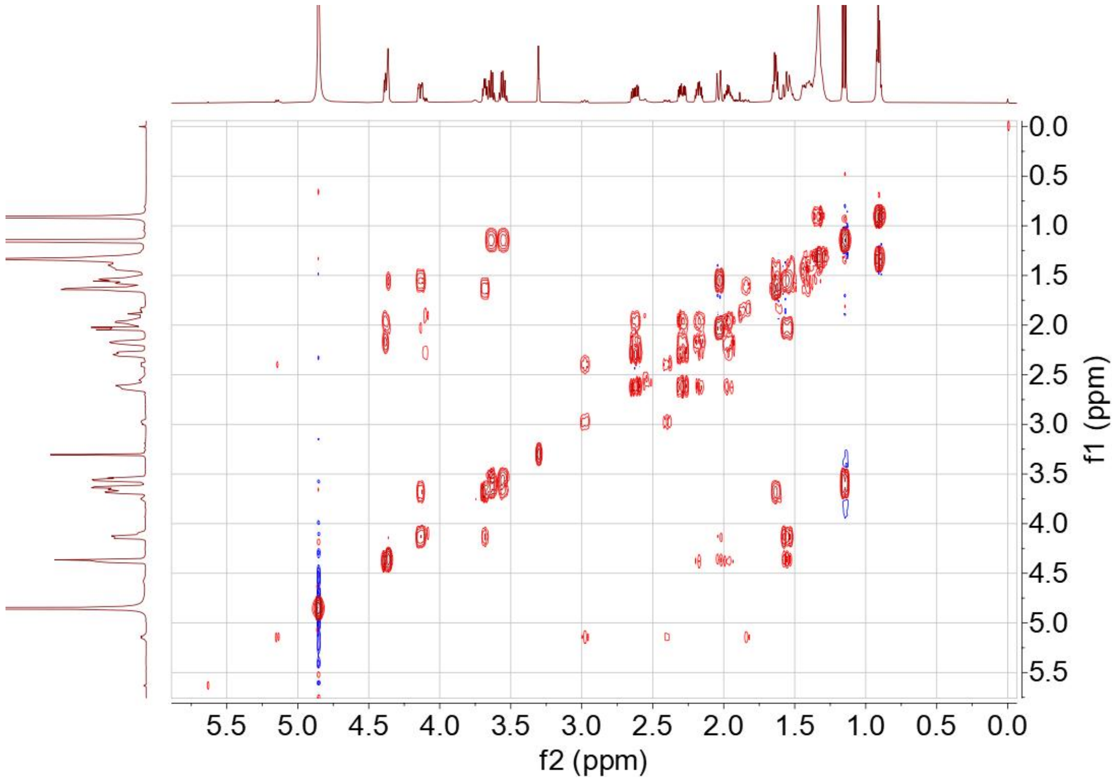

Figure S31. ^{1}H-^{1}H COSY spectrum of compound **3** recorded in CD_3OD

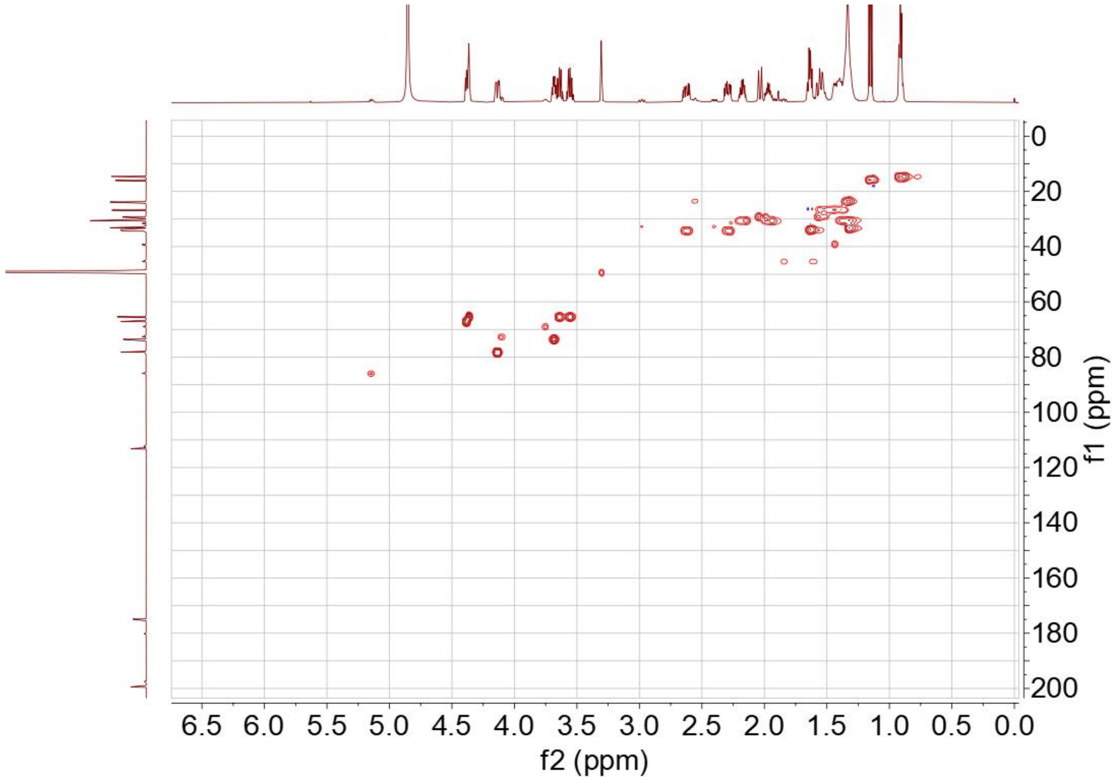

Figure S32. HSQC spectrum of compound **3** recorded in CD_3OD

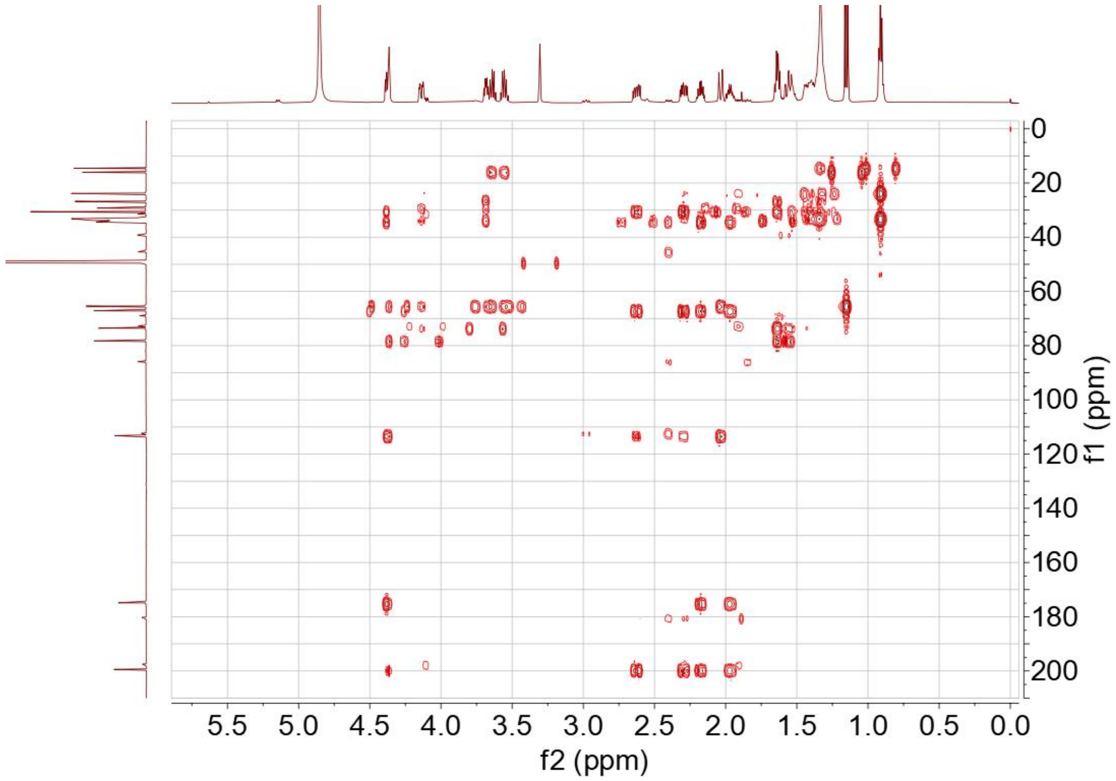

Figure S33. HMBC spectrum of compound **3** recorded in CD_3OD

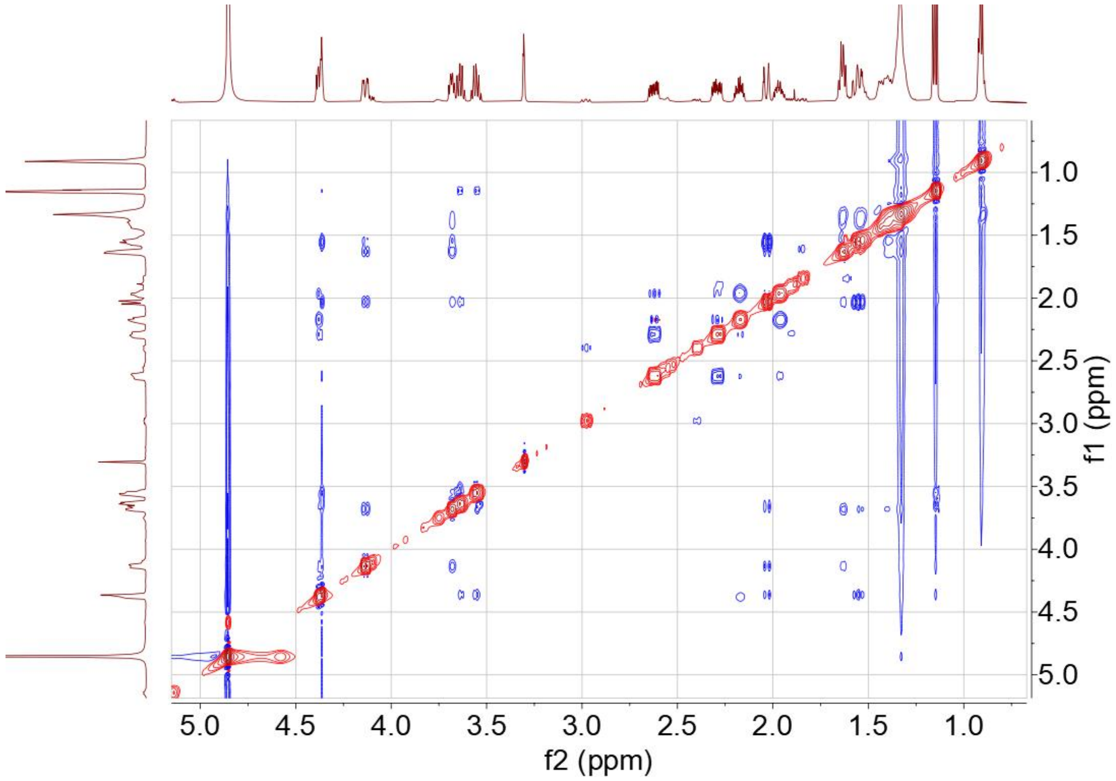

Figure S34. NOESY spectrum of compound **3** recorded in CD_3OD

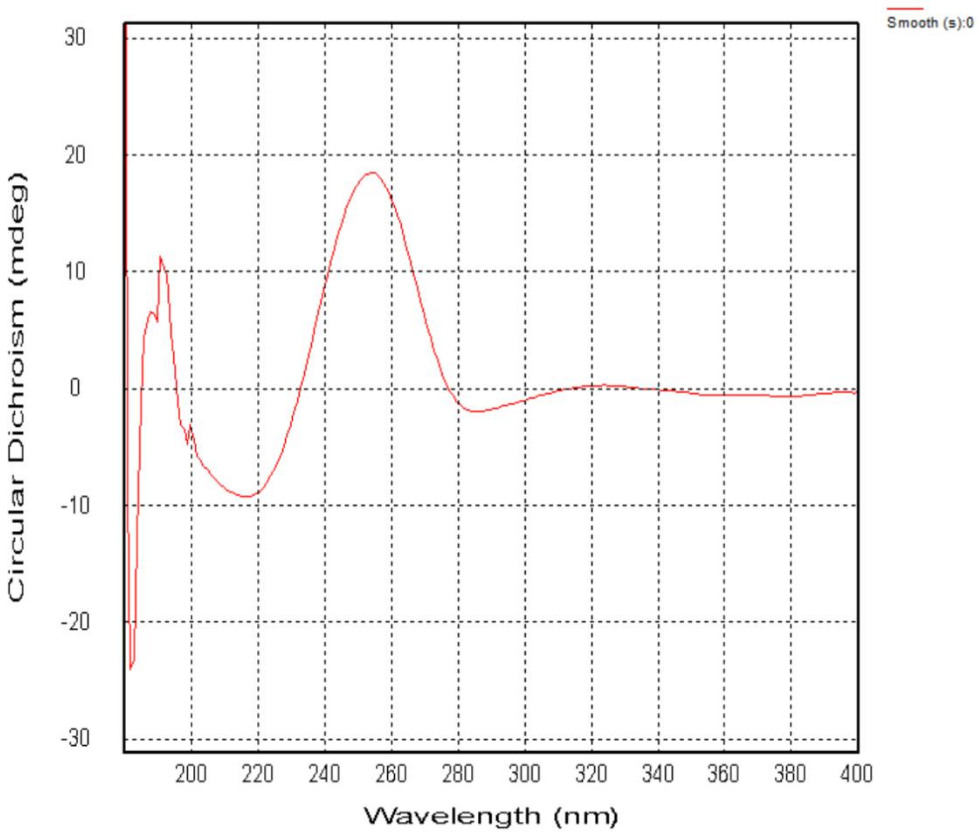

Figure S35. CD spectrum of compound **3**

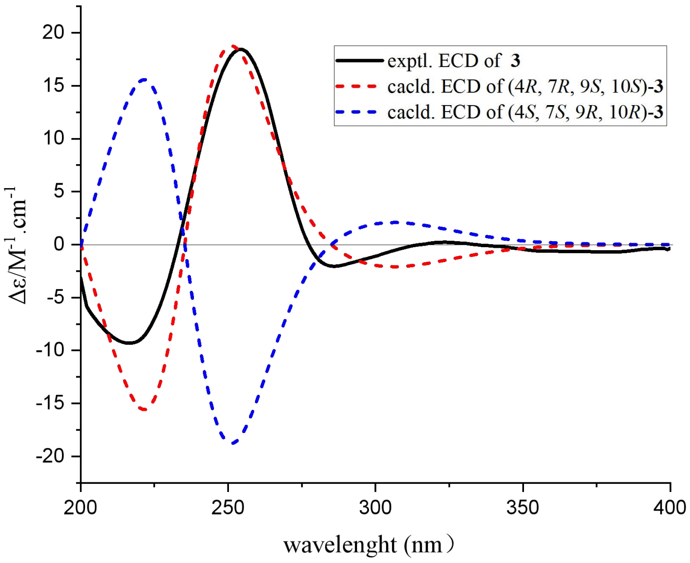

Figure S36. Experimental and calculated ECD spectra of compound 3
